# Sex-specific neural responses to acute psychosocial stress in depression

**DOI:** 10.1038/s41398-021-01768-y

**Published:** 2022-01-10

**Authors:** Daifeng Dong, Maria Ironside, Emily L. Belleau, Xiaoqiang Sun, Chang Cheng, Ge Xiong, Lisa D. Nickerson, Xiang Wang, Shuqiao Yao, Diego A. Pizzagalli

**Affiliations:** 1 Medical Psychological Center, The Second Xiangya Hospital of Central South University, Changsha, Hunan P.R. China; 2 China National Clinical Research Center for Mental Disorders (Xiangya), Changsha, Hunan P.R. China; 3 McLean Hospital, Belmont, MA USA; 4 Harvard Medical School, Boston, MA USA; 5 Laureate Institute for Brain Research, Tulsa, OK USA

**Keywords:** Human behaviour, Depression

## Abstract

Major Depressive Disorder (MDD) is characterized by increased stress sensitivity. Emerging findings in healthy adults suggest that stress responses within limbic/striatal-prefrontal regions are moderated by sex and unfold over time. Thus, we hypothesized that stress response abnormalities in MDD might be affected by sex and stress exposure time. The Montreal Imaging Stress Task was administered to 124 unmedicated patients with first-episode MDD (76 females) and 243 healthy controls (HC; 137 females) during functional magnetic resonance imaging (fMRI). Based on prior studies, amygdala, hippocampus, medial orbitofrontal cortex (mOFC), nucleus accumbens (NAc) and dorsolateral prefrontal cortex (dlPFC) were selected as a priori regions of interest. In a complementary approach, we probed the effects of stress on the frontoparietal network (FPN) and a network including the amygdala, NAc and anterior cingulate cortex (ACC). Across groups, males exhibited higher dlPFC activity and right FPN amplitude than females. Relative to female HCs, the female MDD group had less deactivation in limbic/striatal regions (amygdala, NAc, hippocampus, Amygdala-NAc-ACC network). Furthermore, unlike female HCs, the female MDD group failed to show a significant increase of deactivation over stress exposure time in the amygdala, mOFC and NAc. Our findings confirm the importance of considering sex differences when investigating neural stress responses. Case-control differences in neural stress responses observed in females (but not males) provide insights into sex differences in the etiology and pathophysiology of depression. The failure to deactivate limbic/NAc regions in depressed females point to dysfunction of adaptive stress responses over stress exposure time.

## Introduction

Major depressive disorder (MDD) is a prevalent disorder characterized by high morbidity, recurrence, and rate of suicide. Stress has been strongly implicated in the onset, maintenance and relapse of MDD [1, 2], and increased stress sensitivity has emerged as one of the most promising endophenotypes in MDD [3]. Critically, meta-analytic results demonstrate that women are approximately twice as likely to be diagnosed with MDD than men [4], highlighting that sex is a critical factor in the etiology and pathophysiology of MDD [5]. Thus, investigating the underlying neural mechanisms of stress responses in MDD within the lens of sex differences could provide key insights. Prior neuroimaging studies [6–8] revealed that depressed patients exhibited atypical neural responses to acute stress in limbic (i.e., amygdala, hippocampus) and striatal-prefrontal regions. However, to our knowledge, the potential influence of sex on neural stress responses in depression has been insufficiently evaluated.

Directly relevant to the current study, accumulating neuroimaging findings in healthy adults have uncovered sex differences in neural stress responses [9–15], predominantly in limbic and striatal-prefrontal regions. Although the patterns of findings were mixed, the most common outcome was that men had higher prefrontal responses during stress than women [10, 11, 13, 15], whereas women had higher responses in limbic-striatal regions than men [11, 15]. In light of this evidence, we speculated that neural stress abnormalities in limbic-striatal-prefrontal regions in MDD might be modulated by sex.

Notably, mounting evidence indicates that the time course of stress exposure can affect neural responses [16, 17]. Specifically, a recent study in healthy adults described increasing deactivation in limbic-paralimbic regions (e.g., amygdala, hippocampus, medial prefrontal cortex) throughout a sustained psychosocial stress exposure. Although the functional significance of such deactivation needs to be fully elucidated, these temporal findings indicate that it is pivotal to consider stress exposure time in imaging research [16]. Such temporal effects provide a novel perspective to probe neural stress processing in MDD. To our knowledge, most studies in MDD have not considered the effect of stress exposure time course. Given the overlap of brain regions implicated in stress exposure effects and stress responses in MDD, we hypothesized that MDD and healthy controls would show differential unfolding of stress-related neural responses over time, particularly in limbic-paralimbic regions.

To test these hypotheses, we used the Montreal Imaging Stress Task (MIST) to elicit psychosocial stress [18, 19]. The stress elements of the MIST include social evaluation threat and uncontrollability, which have been found to induce robust stress responses [20]. Of note, various types of stress paradigms (e.g., psychosocial vs. physiological) may evoke different neural processes [21, 22]. In heathy controls, the MIST has been consistently found to deactivate the hippocampus, amygdala, medial orbitofrontal cortex (mOFC), and nucleus accumbens (NAc) [19]. Notably, the hippocampus, amygdala, and mOFC are regions critically implicated in stress processing [23–25] whereas the NAc has emerged as a core region contributing to active coping under stress, as well as interactions between stress and reward processing [6, 25–28]. In addition to these bottom-up stress processing regions, top-down control under stress is important to consider. The dorsolateral prefrontal cortex (dlPFC), a core region of the frontoparietal network (FPN) [29], has been regarded as a crucial region subserving cognitive control. Accordingly, in light of prior findings in healthy controls, five limbic-paralimbic-striatal-prefrontal regions (i.e., amygdala, hippocampus, NAc, mOFC, dlPFC) were included as regions of interest (ROIs) to examine both bottom-up and top-down circuits. To complement a priori ROI analyses, independent component analysis (ICA) was used to identify two functional networks hypothesized to show stress-related effects: a top-down network circuit (FPN) and a bottom-up network circuit that spanned the amygdala, NAc, mOFC.

In sum, in light of (1) sex-specific neural stress responses in limbic-striatal-prefrontal cortex in healthy adults; (2) increased deactivation over stress exposure time in limbic-paralimbic regions in healthy adults; and (3) abnormal neural stress response in limbic-striatal-prefrontal regions in MDD, we hypothesized that both sex and stress exposure time would modulate the neural stress responses in limbic-striatal-prefrontal regions in MDD.

## Materials and methods

### Participants

Patients meeting DSM-IV-TR Axis I Disorders criteria for their first MDD episode were recruited, with exclusion criteria for potential confounding effects of antidepressant medications, multiple episodes and comorbidities. See “Supplementary Methods” for detailed eligibility criteria. All participants were aware of the study’s purpose and provided informed written consent. Ten healthy subjects and 2 depressed patients were excluded because of excessive head movement (see fMRI Preprocessing for exclusion criteria), leaving 124 MDD patients and 243 healthy controls (HCs) available for analyses. Clinical and demographic characteristics of MDD and HCs are summarized in Table 1.

**Table 1:** Demographic and clinical characteristics of unmedicated first-episode MDD and healthy controls.

Characteristics	HC Male (*N* = 106)		HC Female (*N* = 137)		MDD Male (*N* = 48)		MDD Female (*N* = 76)		Diagnosis		Sex		Diagnosis × Sex	
	Mean	SD	Mean	SD	Mean	SD	Mean	SD	F	*P*	F/t	*P*	F	*P*
Age (Years)	20.54	2.13	21.33	4.28	24.75	5.16	25.68	7.90	59.07	<0.001	2.39	0.120	0.02	0.898
Education (Years)	14.20	1.25	14.48	1.61	14.56	1.97	14.03	2.32	0.05	0.827	0.40	0.530	4.31	0.039
Mean FD	0.10	0.04	0.12	0.05	0.10	0.08	0.12	0.06	1.34	0.249	6.15	0.014	0.04	0.846
Illness duration (Months)					8.32	9.41	8.07	9.98	–	–	−0.11	0.910	–	–
HAMD	–	–	–	–	20.35	6.02	22.49	4.69	–	–	−2.21	0.029	–	–
BDI-II	5.53	6.04	5.26	4.89	27.10	10.39	29.65	9.88	776.53	<0.001	1.91	0.168	2.93	0.088
Cognitive	1.82	2.13	1.85	1.90	8.27	3.99	9.19	3.85	482.30	<0.001	2.28	0.132	2.00	0.159
Affective & Somatic	2.30	2.88	1.83	2.09	11.15	4.92	12.57	4.01	721.02	<0.001	1.70	0.19	6.79	0.010
STAI-S	37.02	9.08	37.98	7.99	57.47	13.64	58.42	10.67	345.47	<0.001	0.76	0.386	<0.001	0.996

*FD* framewise displacement, *HAMD* 17 item Hamilton depression rating scale, *BDI-II* Beck depression inventory, *STAI* state and trait anxiety inventory, *HC* healthy controls, *MDD* major depressive disorder.

### Montreal imaging stress task

The MIST is a well-validated acute psychosocial stressor that has been adapted for use with fMRI [8, 18, 19, 30]. For the current study, it was conducted using a block design with three 7-min imaging runs. Each run consisted of three conditions: a rest condition (30 s) with no task requirement; a control condition (90 s) in which the participants answered arithmetic questions with no time limit; and a stressful condition (90 s) in which subjects answered arithmetic questions with a time limit and a visible performance bar. See “Supplementary Methods” (Supplementary Fig. S1) for detailed paradigm design.

### Stress response measurement

Self-report subjective stress ratings and cortisol levels were collected to evaluate stress responses. Levels of subjective stress were assessed immediately before and after the MIST task using a 0–10 visual analog scale (0, no stress; 10, maximum stress). Saliva samples were collected upon participants’ arrival (t = −75 min), after 30-minutes rest (t = −45 min), after entering the scanner (t = −15 min), after 15-minutes anatomical and resting-state scans (t = 0 min), after each MIST run (3runs; t = +7/14/21 min post-stress), and after leaving the scanner (t = +50 min post-stress). See “Supplementary Methods” for details of collection.

### fMRI data acquisition

Scanning was conducted on a 3 T Siemens Magnetom Skyra scanner (Siemens Healthineers, Erlangen, Germany). Blood oxygen level-dependent data were collected with an echo-planar imaging sequence with the following parameters: repetition time/echo time = 2000/30 ms, thickness/gap = 4/1 mm, field of view = 256 mm^2^, flip angle = 80°, matrix = 64 × 64, slices = 32. T1-weighted structural images were acquired with a magnetization-prepared rapid gradient echo with the following parameters: repetition time/echo time = 1900/2.01 ms, thickness/gap = 1/0 mm, field of view = 256 × 256 mm, flip angle = 9°, matrix = 256 × 256, slices = 176.

### fMRI preprocessing

Preprocessing was performed using fMRIPrep 1.5.8 [31] which is based on Nipype 1.4.1 [32]. The BOLD time-series (including slice-timing correction) were resampled into MNI space. Motion artifacts were identified using independent component analysis (ICA-AROMA, [33]) and subsequent visual inspection of ICA components was performed using regfilt inbuilt in FMRIB Software Library Package (FSL) on the preprocessed BOLD time-series in MNI space after removal of non-steady volumes (first 4 volumes) and spatial smoothing with an isotropic, Gaussian kernel of 6 mm full-width half-maximum. Lastly, the denoised bold runs were temporally filtered using a high bandpass of 180 s. Subjects were excluded if they had >20% trials with 0.5 mm movement based on framewise displacement (FD) and/or 1.5 standard temporal derivative of timecourses of RMS variance over voxels. See “Supplementary Methods” for more details.

### Region of interest analyses

**Fig. 1:**
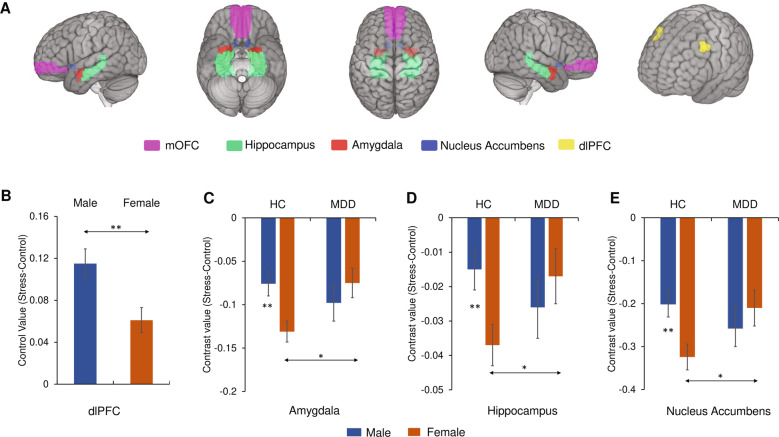
Neural responses to acute psychosocial stress. **A** Location of medial orbitofrontal cortex, hippocampus, amygdala, nucleus accumbens, dorsolateral prefrontal cortex. **B** Sex differences in dorsolateral prefrontal cortex. **C** Sex × Diagnosis interaction effect in amygdala. **D** Sex × Diagnosis interaction effect in hippocampus. **E** Sex × Diagnosis interaction effect in nucleus accumbens. Estimated-mean are plotted, and error bars represents standard error (SE). mOFC medial orbitofrontal cortex, dlPFC dorsolateral prefrontal cortex, HC healthy controls, MDD major depressive disorder. *p_Bonferroni_ < 0.05, **p_Bonferroni_ < 0.01.

The hippocampus, amygdala, and mOFC masks were extracted from the automated anatomical labeling (AAL) atlas 2 inbuilt in the SPM Wake Forest University (WFU) PickAtlas toolbox version 3.0.5b. The NAc mask was extracted from the Harvard-Oxford subcortical atlas (probability threshold, 25%); the dlPFC mask was derived from a meta-analysis of cognitive emotion regulation [34]. See Fig. 1A for the ROI mask locations.

For the first-level analyses, a general linear model including rest, control, and stress conditions was conducted for each participant using Statistical Parametric Mapping (SPM12; The Wellcome Centre for Human Neuroimaging, London, UK). For each of the 5 ROIs, contrast values (stress vs. control) were extracted (5 ROIs × right/left hemisphere × 3 runs). The uncorrected whole-brain *t*-map (stress vs. control) for each group and the detailed contrast values of each ROI for each group can be seen in Supplementary Fig. S2. Consistent with prior findings, the amygdala, NAc, hippocampus, mOFC were deactivated in the stress condition vs. control condition across groups. For the group-level analyses, a repeated-measures MANCOVA was first conducted on four ROIs previously found to show deactivation during acute psychosocial stress (amygdala, NAc, hippocampus, mOFC); Time (run1, run2, run3) and Hemisphere (left, right) were included as additional within-subject factors, while *Sex* and *Diagnosis* (HC, MDD) were included as between-subject factors, and age was included as a covariate. Age (centered) was included as a covariate in all group-level analyses because the MDD group were significantly older than the HCs (25.32 ± 6.96 vs. 20.98 ± 3.52, *p* < 0.001). Significant MANCOVA effects were followed-up with Hemisphere × Time × Sex × Diagnosis ANCOVAs on contrast values for the 4 ROIs. For the dlPFC, an analogous Hemisphere × Time × Sex × Diagnosis ANCOVA was conducted. For statistical power consideration, see the “Supplementary Methods” for details.

### Independent component analysis

To complement ROI analyses, ICA was conducted. The Multivariate Exploratory Linear Optimized Decomposition into Independent Components (MELODIC) software inbuilt in FSL was used to run ICA. Using MELODIC, a group-average concatenated ICA was performed on all subjects (243 HCs, 124 MDD) to get the group-average spatial maps. The dimensionality was set between 10 and 40 in steps of 5 to evaluate an appropriate dimension that captures the brain activation pattern well and also displays the networks of interest clearly. A total of 35 components were finally confirmed based on careful visual inspection on the group ICA spatial maps.

Dual regression was used to extract the individual-specific spatial maps and their corresponding timeseries [35, 36]. First, the group spatial maps of 35 components from group-average concatenated ICA were normalized for the use of dual regression. Second, the normalized group-average spatial maps were regressed into individuals’ 4D dataset to obtain a set of timeseries. Finally, these timeseries were regressed into the same 4D dataset to extract a subject-specific set of spatial maps of 35 components. In summary, the dual regression resulted in a set of spatial maps of 35 components and its corresponding 35 timeseries for each task session. The timeseries of networks of interest were then loaded using a custom Matlab script based on FSLNets v0.6 (https://fsl.fmrib.ox.ac.uk/fsl/fslwiki/FSLNets) for the following statistical analyses.

In light of the hypothesized brain function of some of the selected ROIs (e.g., DLPFC: top-down cognitive control) and replicated findings of stress-related deactivation in the four limbic-paralimbic-striatal regions (mOFC, amygdala, hippocampus, NAc), the FPN (left and right) and an amygdala-NAc-anterior cingulate cortex (ACC) network (hereafter referred to as Amygdala-NAc-ACC network) were selected. A general linear model (GLM) was used to explore the modulation effect of task design on the intra-network amplitude using FSL (*fsl_glm* function). First of all, we convolved the task regressor by double gamma hemodynamic response function (HRF, stress vs. control) using the GLM setup module built in FSL. Since the task design of each task session is identical, the task regressor could be applied to all subjects. Second, for each task session, a GLM with the a timeseries of network as the dependent variable and task regressor as the independent variable was conducted for each network of interest. The beta value of the task regressor was extracted as an index of the modulation effect of task design on network amplitude. After this step, nine beta values [three sessions (run1, run2, run3) × 3 networks of interest] were obtained for each subject. Finally, for group-level analysis, Time × Sex × Diagnosis ANCOVA was run on contrast values of each network.

## Results

### Stress manipulation check

**Fig. 2:**
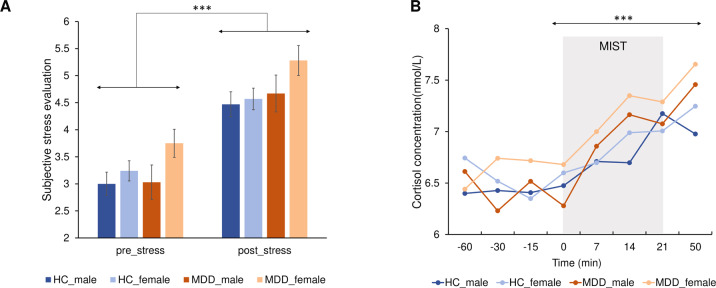
Subjective and cortisol stress responses. **A** All four groups exhibited higher post-MIST subjective stress evaluation level in comparison to the pre-MIST subjective stress level. Estimated-mean are plotted, and the error bar represent SE. **B** Significant main effect of time in cortisol concentration over the stress exposure. ***p_Bonferroni_ < 0.001. HC healthy controls, MDD major depressive disorder, MIST Montreal Imaging Stress Task.

A Time × Diagnosis × Sex repeated-measures ANCOVA revealed a significant main effect of *Time* on self-report responses to stress (F(1,362) = 151.87, *p* < 0.001, *η*^*2*^ = 0.296; Fig. 2A) and cortisol (F(7,1127) = 15.07, *p* < 0.001, *η*^*2*^ = 0.051; 8 timepoints; Fig. 2B), with higher post-MIST subjective stress level (*p*_Bonferroni_ < 0.001) and cortisol concentration (T50 vs. T0, *p*_Bonferroni_ < 0.001), which indicate the MIST successfully elicited psychosocial stress. No significant group effects emerged for cortisol and self-reported stress ratings (*ps* > 0.05; Supplementary Table S1). See “Supplementary Methods” and “Results” for analyses assessing the group effects on stress responses.

### ROI results

#### Main effect of sex

The Hemisphere × Time × Sex × Diagnosis ANCOVA in dlPFC revealed a significant main effect of *Sex* (male > female; F(1,362) = 8.43, *p* = 0.004, *η*^*2*^ = 0.023; Fig. 1B).

#### Diagnosis × sex interaction

A significant Diagnosis × Sex interaction effect emerged for the amygdala (F(1,362) = 5.76, *p* = 0.017, *η*^*2*^ = 0.016), hippocampus (F(1,362) = 4.73, *p* = 0.030, *η*^*2*^ = 0.013) and NAc (F(1,362) = 4.76, *p* = 0.030, *η*^*2*^ = 0.013) (Supplementary Table S2). Bonferroni-corrected simple effects analyses clarified that the female MDD group had significantly less deactivation relative to the female HC group in the amygdala (*p*_Bonferroni_ = 0.011), hippocampus (*p*_Bonferroni_ = 0.032) and NAc (*p*_Bonferroni_ = 0.030) (Fig. 1C–E), whereas the male MDD and HC groups did not differ (*ps*_Bonferroni_ > 0.36). Moreover, the male HC showed significantly less deactivation in comparison to female HC in the amygdala (*p*_Bonferroni_ = 0.003), hippocampus (*p*_Bonferroni_ = 0.007) and NAc (*p*_Bonferroni_ = 0.007) (Fig. 1C–E).

#### Time × diagnosis × sex interaction

**Fig. 3:**
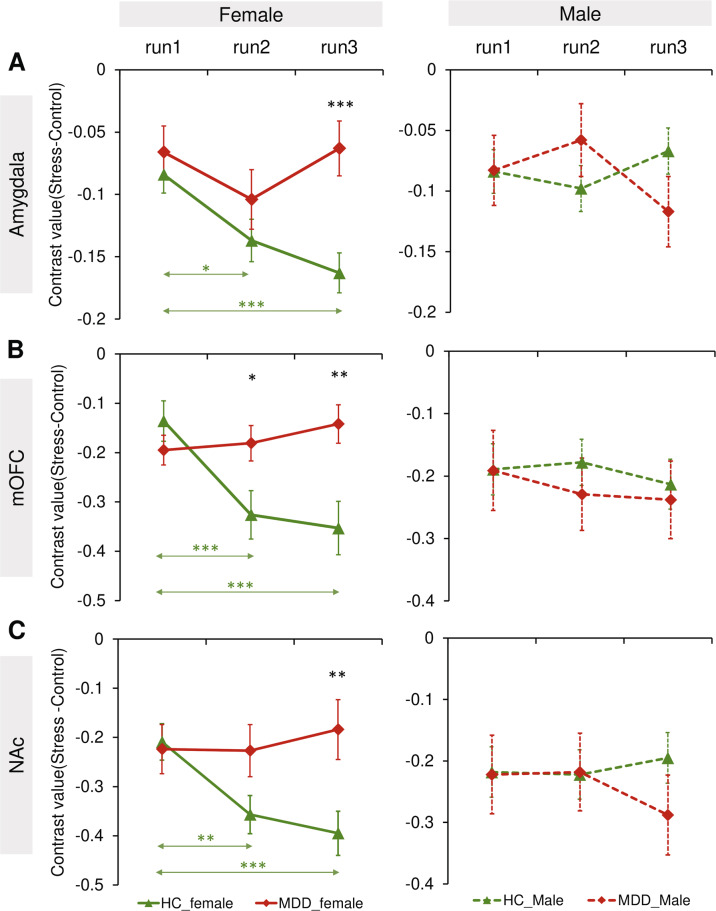
Neural stress responses in different runs. A significant Time × Diagnosis × Sex interaction effect emerged in the (**A**) amygdala, (**B**) medial orbitofrontal cortex, and (**C**) nucleus accumbens. Estimated-mean are plotted, and error bar represent SE. HC healthy controls, MDD major depressive disorder. *p_Bonferroni_ < 0.05, **p_Bonferroni_ < 0.01, ***p_Bonferroni_ < 0.001.

A significant Time × Diagnosis × Sex interaction effect was observed for the amygdala (F(2,724) = 6.44, *p* = 0.002, *η*^*2*^ = 0.017), mOFC (F(2,724) = 4.62, *p* = 0.010, *η*^*2*^ = 0.013), and NAc (F(2,724) = 4.77, *p* = 0.009, *η*^*2*^ = 0.013) (Supplementary Table S2). Owing to these effects, separate Time × Diagnosis ANCOVAs were run for each sex. For females—but not males—significant Time × Diagnosis interactions emerged for the amygdala (F(2,420) = 4.12, *p* = 0.017, *η*^*2*^ = 0.019), mOFC (F(2,420) = 8.08, *p* < 0.001, *η*^*2*^ = 0.037), and NAc (F(2,420) = 4.83, *p* = 0.008, *η*^*2*^ = 0.022) (Fig. 3A–C). In addition, a main effect of Diagnosis for the mOFC (MDD > HC; F(1,210) = 4.63, *p* = 0.033, *η*^*2*^ = 0.033) and amygdala (MDD > HC; F(1,210) = 5.22, *p* = 0.023, *η*^*2*^ = 0.024) was only found in females.

Bonferroni-corrected simple-effect analyses of the Time × Diagnosis interaction effects observed in females revealed that healthy females were characterized by a significant increase of deactivation over stress exposure time in the amygdala (run2 < run1, *p*_Bonferroni_ = 0.015; run3 < run1, *p*_Bonferroni_ < 0.001), mOFC (run2 < run1, *p*_Bonferroni_ < 0.001; run3 < run1, *p*_Bonferroni_ < 0.001) and NAc (run2 < run1, *p*_Bonferroni_ = 0.004; run3 < run1, *p*_Bonferroni_ < 0.001) (Fig. 3A–C). In contrast, MDD females showed no stress-related increase of deactivation over stress exposure time in these regions (*ps*_Bonferroni_ > 0.05). In addition, among females, a significant effect of Diagnosis was observed in run3 in the amygdala (*p*_Bonferroni_ < 0.001), mOFC (*p*_Bonferroni_ = 0.002) and NAc (*p*_Bonferroni_ = 0.007) (Fig. 3A–C), with the MDD group showing less deactivation. For the mOFC, a significant effect of Diagnosis was also observed in run2 in females, with the MDD group showing less deactivation (*p*_Bonferroni_ = 0.020; Fig. 3B).

To further evaluate the robustness of these findings, females were categorized according to whether stress-related deactivation was observed (i.e., run3 – run1 contrast > 0 vs. run3 – run1 contrast < 0). For healthy females (Supplementary Table S3), binomial statistics revealed that the majority of participants showed deactivation for the mOFC (84 of 137, binomial *p*(84/137) = 0.002), amygdala (87 of 137, binomial *p*(87/137) = 0.0004), and NAc (78 of 137, binomial *p*(78/137) = 0.018). For depressed females, opposite patterns were seen since <50% of the sample showed stress-related deactivation for the mOFC (35 of 76, binomial *p*(35/76) = 0.072), amygdala (34 of 76, binomial *p*(34/76) = 0.060), and NAc (30 of 76, binomial *p*(30/76) = 0.017). Chi-square tests confirmed significant differences between female HC and MDD in the proportion of stress-related deactivation in the mOFC (χ² = 4.02, *p* = 0.044), amygdala (χ² = 6.27, *p* = 0.012) and NAc (χ² = 5.28, *p* = 0.021).

See “Supplementary Results” for MANCOVA findings (Supplementary Table S4) and additional, ancillary ROI findings (Supplementary Fig. S3) not central to our hypotheses.

### ICA results

#### Main effect of sex

**Fig. 4:**
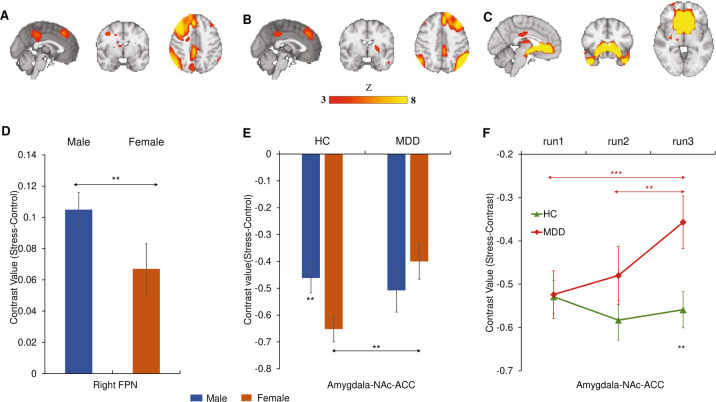
Neural network responses to acute psychosocial stress. Group spatial maps of the (**A**) right frontoparietal network, (**B**) the left frontoparietal network, and (**C**) amygdala-NAc-ACC network. **D** Sex differences in the right frontoparietal network. **E** Sex × Diagnosis interaction effect in the amygdala-NAc-ACC network. **F** Diagnosis × Time interaction effect in the amygdala-NAc-ACC network. Estimated-mean are plotted, and error bars represent SE. HC healthy controls, MDD major depressive disorder, FPN frontoparietal network, NAc nucleus accumbens, ACC anterior cingulate cortex. *p_Bonferroni_ < 0.05, **p_Bonferroni_ < 0.01, ***p_Bonferroni_ < 0.001.

See Fig. 4A–C for the location of three networks of interest. Significant main effect of Sex emerged for network amplitude in the right FPN (males > females, F(1,362) = 9.22, *p* = 0.002, *η*^*2*^ = 0.025; Fig. 4D). No significant main effect of Sex was observed in other networks (*ps* > 0.05; Supplementary Table S5).

#### Diagnosis × sex interaction

A significant Diagnosis × Sex interaction effect was observed in the amygdala-NAc-ACC network (F(1,362) = 5.70, *p* = 0.017, *η*^*2*^ = 0.016). Bonferroni-corrected simple effects analyses showed the female MDD exhibited less deactivation in this network in comparison to female HC (*p*_Bonferroni_ = 0.003; Fig. 4E); in addition, the male HC had less deactivation relative to female HC (*p*_Bonferroni_ = 0.008; Fig. 4E).

#### Time × diagnosis interaction

A significant Time × Diagnosis interaction effect emerged for the amygdala-NAc-ACC network (F(2,724) = 4.67, *p* = 0.010, *η*^*2*^ = 0.013). Bonferroni-corrected simple effects analyses revealed that there was a significant reduction of deactivation (stress minus control) in MDD over stress exposure time (run3 > run1, *p*_Bonferroni_ = 0.006; run3 > run2, *p*_Bonferroni_ = 0.029; Fig. 4F); in addition, MDD patients exhibited significant less deactivation (stress minus control) in run3 relative to HC (*p*_Bonferroni_ = 0.009; Fig. 4F).

### Associations between neural, cortisol and questionnaire measures

See “Supplementary Methods” and Results (Supplementary Table S6–S10 and Supplementary Fig. S4).

## Discussion

The overarching goal of the current study was to test the potential interaction among MDD diagnosis, sex, and the timing of stress exposure during acute psychosocial stress. The fMRI findings revealed that, across diagnostic groups, males exhibited increased neural stress responses in the dlPFC (ROI analysis) and right FPN (ICA analysis) relative to females; moreover, relative to male HCs, female HCs had increased deactivation in the amygdala, hippocampus, NAc, and amygdala-NAc-ACC network. In addition, case-control differences were observed in females (but not males), with less deactivation in limbic-striatal regions including the amygdala, hippocampus, NAc, and amygdala-NAc-ACC network in MDD vs. HC females. Similarly, a Time × Diagnosis interaction effect in the amygdala, mOFC, NAc was only observed in females, with the MDD females showing less deactivation over time than the HC females. Collectively, these findings provide novel evidence for sex-specific unfolding of neural responses to acute psychosocial stress in MDD.

Prior studies in healthy controls have clarified that higher dlPFC activity and FPN amplitude in the stress condition is necessary for maintaining good task performance and cognitive emotion regulation [22, 37, 38]. In addition, a wealth of evidence suggests that the amygdala is implicated in threat perception and emotion processing [23], and NAc is considered a critical region associated with reward and saliency processing [28]. Accordingly, one explanation for the current findings may be that males—at least in the context of the MIST, which is a performance-based stress manipulation [39]—might be more likely to engage top-down control resources under stress in comparison to females; in contrast, the female HCs may engage more bottom-up resources when conducting stress processing. An alternative interpretation of the higher dlPFC activity in males may be that the males have lower neural efficiency of cognitive control, with more activation required in order to maintain equivalent stress levels. Overall, the current findings uncover possible sex-specific neural engagements when reacting to acute psychosocial stress; these findings highlight the importance of considering sex as an important variable when conducting stress-related neuroimaging research.

Case-control differences in neural stress responses were only observed in females, but not in males. Specifically, the female MDD group showed less deactivation in limbic-striatal regions (i.e., amygdala, hippocampus, NAc, and amygdala-NAc-ACC network) in comparison to the female HCs. Considering that the current study is an achievement-based stress task characterized by high cognitive demand, deactivating regions associated with threat detection and reward sensitivity could be an adaptive way to maintain better performance when facing stress. In support of this point, prior literature in healthy adults using the MIST suggested that individuals with absent/less deactivation of limbic/paralimbic regions under stress reported a more stressful social environment [30], increased depressive symptoms [40] and higher trait anxiety [41]. Thus, the current findings in female MDD may point to a reduced ability to deactivate the bottom-up stress circuit when experiencing acute psychosocial stress.

Sex-specific effects in MDD were further illuminated by our analysis of time-exposure effects. Specifically, a Time × Diagnosis interaction effect in the amygdala, mOFC, and NAc emerged only for females. Healthy—but not depressed—females were characterized by increased deactivation over stress exposure time in the amygdala, NAc, and mOFC. Postulating that the deactivation of limbic-paralimbic-striatal regions in the stress condition in the MIST is adaptive, the failure to deactivate the limbic-paralimbic-striatal regions in female MDD may point to a dysfunction of adaptive stress processing over the exposure time. In addition, the absence of this time-related neural pattern in female MDD may also imply a new angle to uncover the abnormal stress processing of depression. In line with this interpretation, one fMRI study also found an increased deactivation in limbic/paralimbic regions in healthy adults over stress-exposure time [16]. The current study confirmed this relatively novel finding and extended it to a clinically depressed sample. However, it is also possible that this BOLD deactivation pattern could be explained by stress habituation. Future work is needed to tease apart which mechanisms might be driving this stress-induced limbic/paralimbic deactivation.

When considering an ICA-based approach, we further found that the MDD group (across both sexes) exhibited decreased deactivation in the amygdala-NAc-ACC network over the stress-exposure time, whereas the HCs had stable deactivation of this network over time. Although we observed in both the ROI and ICA findings that females with MDD had less deactivation than female HCs, the expected increased female HC deactivation over stress-exposure time did not emerge in the ICA results. The discrepancy between the ROI and ICA findings may stem from the differences of the analysis approach (region-focus vs. network-focus). Although the amygdala-NAc-ACC network overlaps with included ROIs, this network also incorporates other brain regions, including the ACC and temporal pole, among others (Supplementary Fig. S5). Moreover, prior literature has shown that the ROIs probed here (e.g., amygdala, mOFC) are sex-dimorphic [42, 43]. Hence, the sex-specific effects of stress response could be obscured when investigating it from the system level. Of note, the reduced deactivation of the amygdala-NAc-ACC network in MDD over stress exposure time suggests a common ground across sex in MDD. One potential speculation for this neural pattern observed in MDD is stress sensitization; however, further investigations with a more specific design are needed.

Some limitations of the current study should be addressed. First, as menstrual cycle [10] and estradiol level [44] can affect neural stress responses, future studies should investigate their role in shaping stress responses using standardized tools [45]. Second, the stress condition induced by the MIST is a combination of increased cognitive demand, social evaluative threat, and the processing of failure [46], which may make it is difficult to understand which process and brain regions are specifically associated with the various stress components. Third, with regard to exploring the temporal unfolding of stress responses, a longer duration of stress condition might be useful for future studies to confirm the time-related findings. Moreover, although the repeated-measure ANOVA is an appropriate method for the current analysis, the multilevel growth models could be an alternate way for future confirmation of time-exposure effects [47, 48]. Fourth, affective ratings were only collected before and after the MIST, rather than between the MIST sub-blocks, thus putative time-exposure effects on subjective stress feeling might have been masked. Fifth, our depressed sample is less representative of the community, which may limit the generalization of our findings. Last, although age was included as a covariate when conducting statistical analyses, the unmatched age across diagnosis group is a limitation that should be mentioned. To further addressing the concern of confounding effects of age on diagnosis-related findings in the current research, we compared the Sex × Time × Hemisphere × Age generalized linear model (GLM) and Diagnosis × Sex × Time × Hemisphere × Age GLM (See more details on “Supplementary Methods and Results”) in the amygdala, hippocampus, NAc, mOFC and Amy-NAc-ACC network. The results showed that the inclusion of the Diagnosis variable significantly improved the goodness of fit of the model in terms of the amygdala, NAc, mOFC, and Amy-NAc-ACC network, indicating the diagnosis-related findings in these regions are not driven by age differences. For the hippocampus, the inclusion of the Diagnosis variable did not significantly improve this model (models were equally good), which may point to potential confounding effects of age on the diagnosis-related neural differences in the hippocampus. Although there were no significant correlations between age and average contrast value (stress vs. control) in the hippocampus for each group (See “Supplementary Results”), further replication of the current Diagnosis-related findings in the hippocampus is warranted.

In spite of these limitations, several novel findings emerged. First, observed sex differences in HCs highlight putative sex-specific neural stress response as well as the importance of considering sex differences in stress research. Second, case-control differences in neural stress responses were only observed in females, which may provide evidence for the sex differences in etiology and pathophysiology of depression. Third, the failure to deactivate limbic-paralimbic-striatal regions in depressed females may implicate its dysfunction of adaptive stress responses over the course of stress exposure. Finally, the observed changes in activation over the course of the stressor emphasize the importance of stress trajectory and timing in neuroimaging research.

## Supplementary information

SUPPLEMENTAL MATERIAL
