# Supplementary material for: Sex-specific neural responses to acute psychosocial stress in depression
Source: Transl Psychiatry. 2022 Jan 10;12:2. doi: 10.1038/s41398-021-01768-y (PMC8748634; doi:10.1038/s41398-021-01768-y)
Supplement: Supplementary file 1 — SUPPLEMENTAL MATERIAL [file 41398_2021_1768_MOESM1_ESM.docx]

**Sex-specific Neural Responses to Acute Psychosocial Stress in Depression**

***Supplemental Information***

**Supplemental Methods**

***Participants***

Two psychiatrists conducted psychiatric evaluations using the Structured Clinical Interview for DSM-IV-TR Axis I Disorders–Patient Edition [1]. The Beck Depression Inventory-II [2] and the 17-item Hamilton Depression Rating Scale [3] were administered to evaluate current clinical and depressive symptoms. For both patients with major depressive disorder (MDD) and healthy controls, the exclusion criteria were: 1) prior DSM-IV-TR disorder; 2) history of antidepressant use or psychotherapy; 3) history of alcohol/substance abuse; and 4) neurological disorder diagnosis, structural brain abnormality, or MRI contradiction. The study was conducted in accordance with the Declaration of Helsinki and was approved by the ethics committee of the Second Xiangya Hospital of Central South University.

***Montreal Imaging Stress Task (MIST)***

The MIST is a widely used stress paradigm that has been adapted for functional magnetic resonance imaging. For the current study, the MIST included three sessions, and each session included 3 different conditions (rest, control and stressful). The rest condition (30s) aimed at assessing a baseline state in which the participants were asked to keep their eyes open and not press buttons until the next mental arithmetic task appeared. The control condition (90s) aimed at assessing brain activation associated with the mental arithmetic task without stress components. The participants were asked to try to perform the task as accurately and quickly as possible. In this condition, the average accuracy rate could reach about 90%. Finally, for the stressful condition (90s), time pressure was induced by titrating the time bar to each participant’s performance, to maintain approximately 50% correct rate. A performance bar at the top of the screen showed that the participant’s performance was “below the average subject,” set artificially to 80%. Subjects also received “correct” or “incorrect” feedback after answering each question, as well as scripted negative verbal feedback via headphones.

***Stress Response Measurement***

Self-reported subjective stress ratings and cortisol levels were administrated to evaluate stress responses. The self-reported subjective stress ratings were assessed directly before and after the MIST using a 0-10 visual analog scale (0, no stress; 10, maximum stress). Subjective stress responses were calculated by subtracting the pre-stress level from the post-stress level.

In addition, eight saliva samples were collected in the scanner during the interval of scanning to evaluate the changes of cortisol level over the MIST. Saliva samples were collected upon participants’ arrival (t = -75 min), after 30-minutes rest (t = -45 min), after entering the scanner (t = -15 min), after 15-minutes anatomical and resting-state scans (t = 0 min), after each MIST run (3runs; t = +7/14/21 min post-stress), and after leaving the scanner (t = +50 min post-stress). In order to measure cortisol responses, the area under the curve with respect to ground (*AUC_G_*; index of measuring the overall cortisol output) and the area under the curve with respect to increase (*AUC_I_*; index of assessing the cortisol changes) over the stress exposure [cort4 (t = 0 min) to cort8 (t = +50 min)] were calculated [4, 5]. Both the *AUC_G_* and *AUC_I_* were calculated on the natural log-transformed cortisol concentrations.

***Preprocessing of fMRI data***

Preprocessing was performed using fMRIPrep 1.5.8 [6, 7] which is based on Nipype 1.4.1 [8, 9]. Details of the standardized pipeline below were produced by fMRIPrep.

*Anatomical Data Preprocessing*

T1-weighted (T1w) images were corrected for intensity non-uniformity with N4BiasFieldCorrection [10] distributed with ANTs 3.0.0 [11], and used as T1w-reference throughout the workflow. The T1w-reference was then skull-stripped with a Nipype implementation of the antsBrainExtraction.sh workflow (from ANTs), using OASIS30ANTs as target template. Brain tissue segmentation of cerebrospinal fluid (CSF), white-matter (WM) and gray-matter (GM) was performed on the brain-extracted T1w using fast (FSL 6.0.0, RRID:SCR_002823, [12]). Brain surfaces were reconstructed using recon-all (FreeSurfer 6.0.1, RRID:SCR_001847, [13]), and the brain mask estimated previously was refined with a custom variation of the method to reconcile ANTs-derived and FreeSurfer-derived segmentations of the cortical gray-matter of Mindboggle (RRID:SCR_002438, [14]). Volume-based spatial normalization to two standard spaces (MNI152NLin2009cAsym, MNI152NLin6Asym) was performed through nonlinear registration with antsRegistration (ANTs 3.0.0), using brain-extracted versions of both T1w reference and the T1w template. The following templates were selected for spatial normalization: ICBM 152 Nonlinear Asymmetrical template version 2009c [[15], RRID:SCR_008796; TemplateFlow ID: MNI152NLin2009cAsym], FSL’s MNI ICBM 152 non-linear 6th Generation Asymmetric Average Brain Stereotaxic Registration Model [[16], RRID:SCR_002823; TemplateFlow ID: MNI152NLin6Asym].

*Functional Data Preprocessing*

For each of the three BOLD runs, the following preprocessing was performed. First, a reference volume and its skull-stripped version were generated using a custom methodology of fMRIPrep. A deformation field to correct for susceptibility distortions was estimated based on fMRIPrep’s fieldmap-less approach. The deformation field resulted from the co-registration of the BOLD reference to the same-subject T1w-reference with its intensity inverted [17, 18]. Registration was performed with antsRegistration (ANTs 3.0.0), and the process regularized by constraining deformation to be nonzero only along the phase-encoding direction, and modulated with an average fieldmap template [19]. Based on the estimated susceptibility distortion, a corrected EPI (echo-planar imaging) reference was calculated for a more accurate co-registration with the anatomical reference. The BOLD reference was then co-registered to the T1w reference using bbregister (FreeSurfer) which implements boundary-based registration [20]. Co-registration was configured with six degrees of freedom. Head-motion parameters with respect to the BOLD reference (transformation matrices, and six corresponding rotation and translation parameters) were estimated before any spatiotemporal filtering using mcflirt (FSL 6.0.0, [21]). BOLD runs were slice-time corrected using 3dTshift from AFNI 20190007 ([22], RRID:SCR_005927). The BOLD time-series (including slice-timing correction when applied) were resampled onto their original, native space by applying a single, composite transform to correct for head-motion and susceptibility distortions. These resampled BOLD time-series was referred to as preprocessed BOLD in original space, or just preprocessed BOLD. The BOLD time-series were resampled into MNI152NLin6Asym standard space using antsApplyTransforms (ANTs 3.0.0). Motion artifacts were identified using independent component analysis (ICA-AROMA, [23]) and subsequent visual inspection of ICA components was performed using regfilt (FSL) on the preprocessed BOLD time-series in MNI space (MNI152NLin6Asym) after removal of non-steady volumes (first 4 volumes) and spatial smoothing with an isotropic, Gaussian kernel of 6mm FWHM (full-width half-maximum). Lastly, the denoised bold runs were temporally filtered using a high bandpass of 180 sec. Subjects were excluded if they had more than 20% trials with 0.5mm movement based on framewise displacement (FD) and/or 1.5 standard temporal derivative of timecourses of RMS variance over voxels (DVARS). Ten healthy subjects and 2 depressed patients were excluded because of excessive head movement.

***Power Analysis***

A power analysis was performed to compute the required sample size for the repeated measures MANCOVA using the G*Power software (version 3.1.0.7). An effect size associated with a significant *Sex* × *Diagnosis* interaction (partial *η^2^* = 0.11) from a prior study (22) was used for power analyses. This effect size (partial *η^2^* = 0.11, f = 0.351) was used to calculate the required sample size. Parameters for calculating total sample size were: effect size f = 0.351, α error probability = 0.05, power (1- β error probability) = 0.95, number of groups = 4, number of measurements = 24 (2 hemispheres × 3 timepoints × 4 ROIs). The power analysis revealed that 148 participants were required to achieve a power of 0.95 to observe a significant *Sex* × *Diagnosis* interaction. Thus, our actual sample size (N = 367) is enough for the current research.

***Analysis for exploring group effects on subjective and cortisol stress responses***

A *Sex* × *Diagnosis* repeated-measures ANCOVA was run to assess the between-subject effects on cortisol response (*AUC_g_* and *AUC_i_*) and subjective stress response (post-stress minus pre-stress).

***Correlation analyses***

To further characterize the functional significance of putative findings (e.g., stress-related limbic deactivation) in healthy females, Pearson correlations were run between the deactivation (run3 minus. run1) in amygdala, NAc, mOFC and cortisol responses (*AUC_I_*, *AUC_G_*) in healthy females. Similarly, since we observed a significant decrease of neural deactivation over the stress exposure time in amygdala-NAc-ACC network in MDD, Pearson correlations were computed between the reduction of deactivation (run3 minus. run1) and cortisol responses (*AUC_I_*, *AUC_G_*) as well as depressive symptoms (BDI score) in MDD. Finally, Pearson correlations were run between the average contrast values (stress vs. control; the average of three runs) of each region (5 ROIs) and network of interest (3 networks) and depressive symptoms (BDI score) in MDD. In light of sex-specific neural stress responses emerging from the current study, the correlation analyses were conducted in each sex separately.

In addition, with the aim of exploratorily detecting the potential correlations between all variables of interest, Pearson correlation analysis among all variables (average contrast values of each region (5 ROIs), average contrast values of each network of interest (3 networks), amygdala(run3-run1), NAc(run3-run1), mOFC(run3-run1), amygdala-NAc-ACC network (run3-run1), BDI score, AUC_g_, AUC_i_, mean FD, Age) for each group (HC/male, HC/female, MDD/male MDD/female).

***Model Comparison***

To investigate the confounding effects of age on the observed Diagnosis-related neural findings in amygdala, hippocampus, NAc, mOFC and amygdala-NAc-ACC network, we compared the goodness of fit between the *Sex* × *Time* × *Hemisphere* × *Age* generalized linear model (GLM) and *Sex* × *Diagnosis* × *Time* × *Hemisphere* × *Age* GLM using SPSS 22.0. The effects detected are identical with the full-factorial repeated-measures ANCOVA model used in the current study. The Akaike information criterion (AIC) value, was used for the model selection, which balances goodness of fit with a penalty for additional model terms to ensure most parsimonious model fit. The smaller AIC value indicates better model fit, with a difference of 10 between AICs being considered significant improvement [26].

**Supplemental Results**

***Group effects on subjective and cortisol stress responses***

The ANCOVA on self-reported stress responses (post-stress level minus pre-stress level) did not reveal a main effect of *Sex* (F(1,362) = 0.28, *p* = 0.599), *Diagnosis* (F(1,362) = 0.46, *p* = 0.496) or an interaction effect of *Sex* × *Diagnosis* (F(1,362) = 0.001, *p* = 0.972). Similarly, an ANCOVA analysis of cortisol stress responses (*AUC_I_* and *AUC_G_*) did not reveal a main effect of *Sex* (*AUC_I_*, F(1,309) =1.06, *p* = 0.304; *AUC_G_*, F(1,309) = 0.74, *p* = 0.389), *Diagnosis* (*AUC_I_*, F (1,309) = 1.01, *p* = 0.315; *AUC_G_*, F(1,309) = 0.70, *p* = 0.404) or an interaction effect of *Sex* × *Diagnosis* (*AUC_I_*, F(1,309) = 0.83, *p* = 0.363; *AUC_G_*, F(1,309) = 0.004, *p* = 0.949).

***Supplemental ROI findings***

1. ***MANCOVA analysis in limbic-paralimbic-striatal ROIs***

The MANCOVA on the four limbic-paralimbic-striatal regions revealed two significant effects involving *Diagnosis*: a *Time* × *Diagnosis* × *Sex* interaction (Wilks’ Lambda = 0.970, F (2, 361) = 5.61, *p* = 0.004, *η^2^* = 0.030) and a *Region* × *Time* × *Diagnosis* × *Sex* interaction (Wilks’ Lambda = 0.942, F (6,357) = 3.64, *p* = 0.002, *η^2^* = 0.058). Given these effects, follow-up repeated-measures ANCOVAs analyses were conducted for each ROI separately (Section C). Other effects not involving *Diagnosis* are reported in the Supplemental Table S4.

1. ***dlPFC ROI***

The *Hemisphere* × *Time* × *Sex* × *Diagnosis* ANCOVA revealed a significant *Time* × *Hemisphere* × *Diagnosis* interaction effect for the dlPFC (F (2,724) = 4.64, *p* = 0.010, *η^2^* = 0.013) (Supplemental Table S2). A follow-up *Time* × *Diagnosis* ANCOVA was performed in each hemisphere separately; these analyses revealed a significant *Time* effect for the left dlPFC (run3 > run1, F (2,728) = 7.96, *p* < 0.001, *η^2^* = 0.021; Supplemental Fig.S3); a significant main effect of *Diagnosis* and a significant *Time* × *Diagnosis* interaction effect did not emerge for either hemisphere (*ps* > 0.05). A significant *Time* × *Hemisphere* interaction effect (F (2,724) = 8.22, *p* < 0.001, *η^2^* = 0.022; Supplemental Table S2) emerged, owing to the significant *Time* effects in the left hemisphere (run3 > run1, *p* _Bonferroni_ < 0.001) and significant hemisphere effects on run1(right > left, *p* _Bonferroni_ < 0.001). In addition, a main effect of *sex* also emerged, see details in main text.

1. ***Follow-up analysis***

*Diagnosis × Sex and Diagnosis × Sex × Time interaction effects can be seen in main text.*

*Hemisphere × Sex interaction effect on NAc*

For the NAc, there was a significant *Hemisphere* × *Sex* interaction effect (F(1,362) = 4.96, *p* = 0.027, *η^2^* = 0.014; Supplemental Table S2). Bonferroni-corrected simple effects analyses revealed a trend with the left nucleus accumbens exhibiting less deactivation in comparison to the right nucleus accumbens in males (left hemisphere > right hemisphere, *p* _Bonferroni_ = 0.072) but not in females (*p* _Bonferroni_ = 0.194).

*Effects on mOFC*

A main effect of *Hemisphere* emerged for mOFC (left < right; F(1, 724) = 19.96, *p* < 0.001, *η^2^* = 0.052) and dlPFC (left < right; F (1, 724) = 4.93, *p* = 0.027, *η^2^* = 0.013) (Supplemental Table S2).

For mOFC, a significant *Time* effect (F (2,724) = 3.37, *p* = 0.035, *η^2^* = 0.009) and *Time* × *Diagnosis* interaction effect (F (2, 724) = 3.21, *p* = 0.041, *η^2^* = 0.009) emerged (Supplemental Table S2), owing to the increased deactivation in HCs. Because a significant *Time* × *Diagnosis* × *Sex* interaction effect (F(2,724) = 4.62, *p* = 0.010, *η^2^* = 0.013; Supplemental Table S2) emerged, suggesting the *Time* × *Diagnosis* interaction effect differs across sex. Thus, it is more reasonable to interpret the *Time* × *Diagnosis* interaction in each sex which have been well described in the main text.

***Association between neural, cortisol and questionnaire measures***

Among heathy females, significant negative correlations were observed between the *AUC_G_* and the deactivation (run3 minus run1) in the amygdala (*r* = -0.23, *p* = 0.011) and medial orbitofrontal cortex (*r* = -0.22, *p* = 0.015). In addition, a significant negative association was found between the average contrast value (stress vs. control) of the hippocampus and BDI score in female MDD (*r* = -0.29, *p* = 0.011). No other significant associations were observed (Supplemental Table S6). All reported *p*-values of correlation analysis are uncorrected for multiple comparisons (25 correlation analysis were conducted in total), and should thus interpreted with caution.

For the results of exploratory correlation analysis among all variables per group, see Supplemental Table S7-S10.

***Result of model comparison***

Our results revealed that the GLM model with *Diagnosis* was significantly improved in comparison to the GLM model without *Diagnosis* in terms of the amygdala (AIC, -858.06 vs. -841.01), NAc (AIC, 2743.22 vs. 2755.47), mOFC (AIC, 2255.35 vs. 2267.29), and amygdala-NAc-ACC network (AIC, 2135.61 vs. 2150.51), indicating the diagnosis-related findings in the amygdala, NAc, mOFC and amygdala-NAc-ACC network are not driven by the age differences. With regard to the hippocampus, the GLM model with Diagnosis was not significantly different to the GLM model without Diagnosis (AIC, -4087.86 vs. -4089.08), suggesting potential confounding effects of age on diagnosis-related findings in hippocampus.

**Supplemental Discussion**

Increased deactivation (run3 minus run1) in the amygdala and mOFC over stress exposure time in healthy females was associated with higher cortisol *AUC_G_*, whereas no associations were observed between this and the deactivated amplitude in limbic/paralimbic regions (i.e., amygdala, NAc, mOFC). There were no significant associations between any of these regions and *AUC_I_*. In order to better interpret this association pattern, we categorized the healthy females according to whether stress-related deactivation was observed (i.e., run3 – run1 contrast > 0 vs. run3 – run1 contrast < 0) and plotted the cortisol trajectory of these two groups (see Supplemental Fig. S4). The *AUC_I_* represents the cortisol changes from individual pre-stress baseline, whereas the *AUC_G_* uses zero as a baseline, thus revealing the general magnitude of cortisol concentration and change (for more details see [4]). Thus, the current findings imply that female individuals with a higher general cortisol level are more likely to exhibit adaptive neural responses over the stress exposure time. In support of this possibility, some prior findings also suggested that individuals with higher basal cortisol level showed higher stress resilience (i.e., high extroversion score [27] , higher amygdala-prefrontal connectivity [28]), and lower stress sensitivity (i.e., lower amygdala activation induced by viewing negative emotional faces [27]). In addition, we observed a significant negative association between depressive symptoms and activation in the hippocampus in female MDD, suggesting that female MDD individuals with lower activation in the hippocampus exhibited high severity of depressive symptoms. However, the female HC exhibited lower activation of the hippocampus in comparison to the female MDD group, thus the direction of this association is unexpected. Since the function of the hippocampus in the current stress task (e.g., HPA axis, work memory) is complex, a more specific design is warranted to confirm this finding. Of note, such correlation was relatively weak when considering multiple comparison correction; accordingly, future studies will be needed to confirm its robustness.

**Supplemental Figures**

**
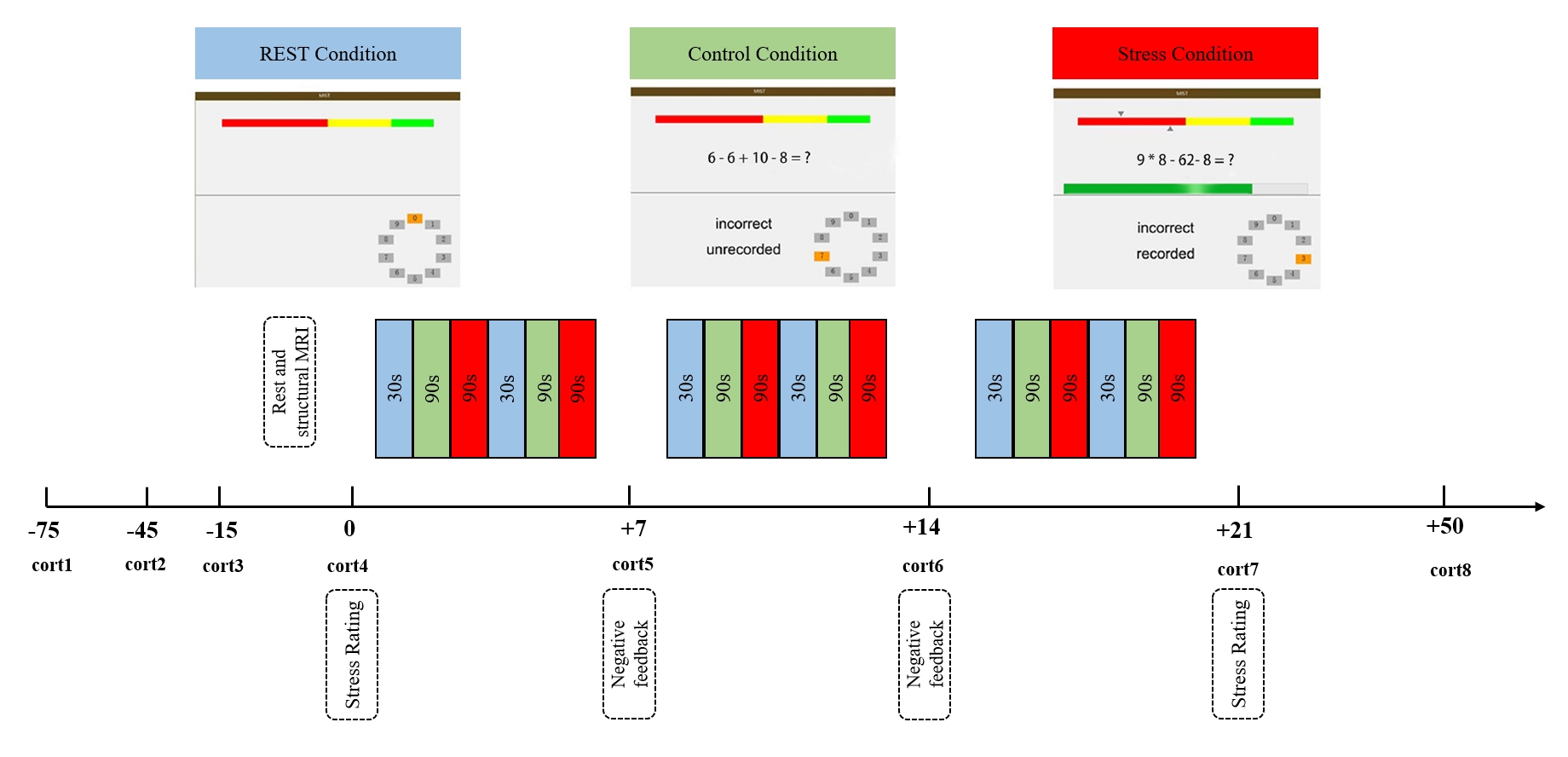
**

**Supplemental Fig. S1. The overview of experimental design.** The MIST included 3 sessions. Each session lasted 7 minutes. Eight saliva samples were collected across the task and subjective stress levels were collected immediately before and after the MIST task. In addition, the participant received scripted negative feedback after the first and the second MIST session. The blue rectangle represents the rest condition in which participants did not have any task requirement; the green rectangle represents the control condition in which participants were asked to answer arithmetic questions without a time limit; the red rectangle represents the stress condition in which participants need to answer questions with the time limit. Cort: cortisol


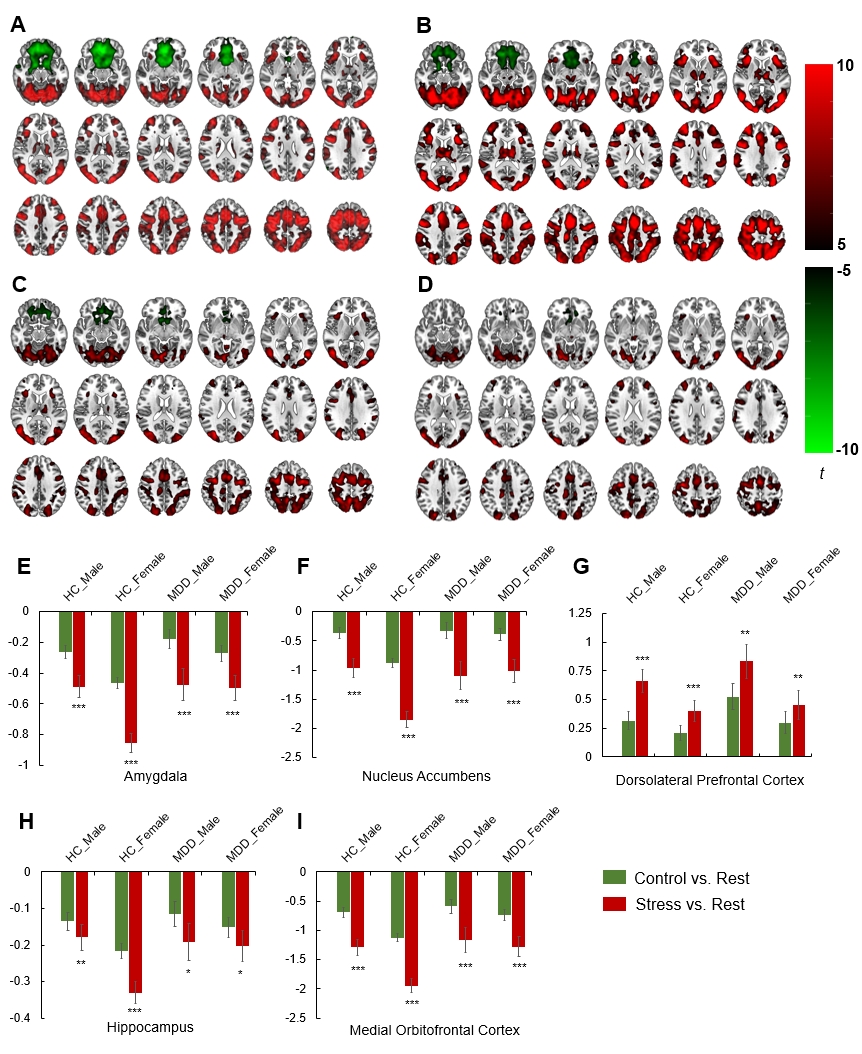


**Supplemental Fig. S2. Neural stress responses of the whole brain and regions of interest.** The neural stress responses (Stress vs. Control) of the whole brain (uncorrected) in **(A)** healthy females, **(B)** healthy males, **(C)** females with depression, and **(D)** males with depression. The green color indicates brain regions that were deactivated in the stress condition in comparison to the control condition; the red color highlights brain regions that were activated in stress condition relative to control condition. The neural stress responses (control vs. rest; stress vs. rest) in the **(E)** amygdala, **(F)** nucleus accumbens, **(G)** dorsolateral prefrontal cortex, **(H)** hippocampus, and **(I)** medial orbitofrontal cortex. HC: healthy controls; MDD: major depressive disorder. ^*^*p* < 0.05, ^**^*p* < 0.01, ^***^*p* < 0.001


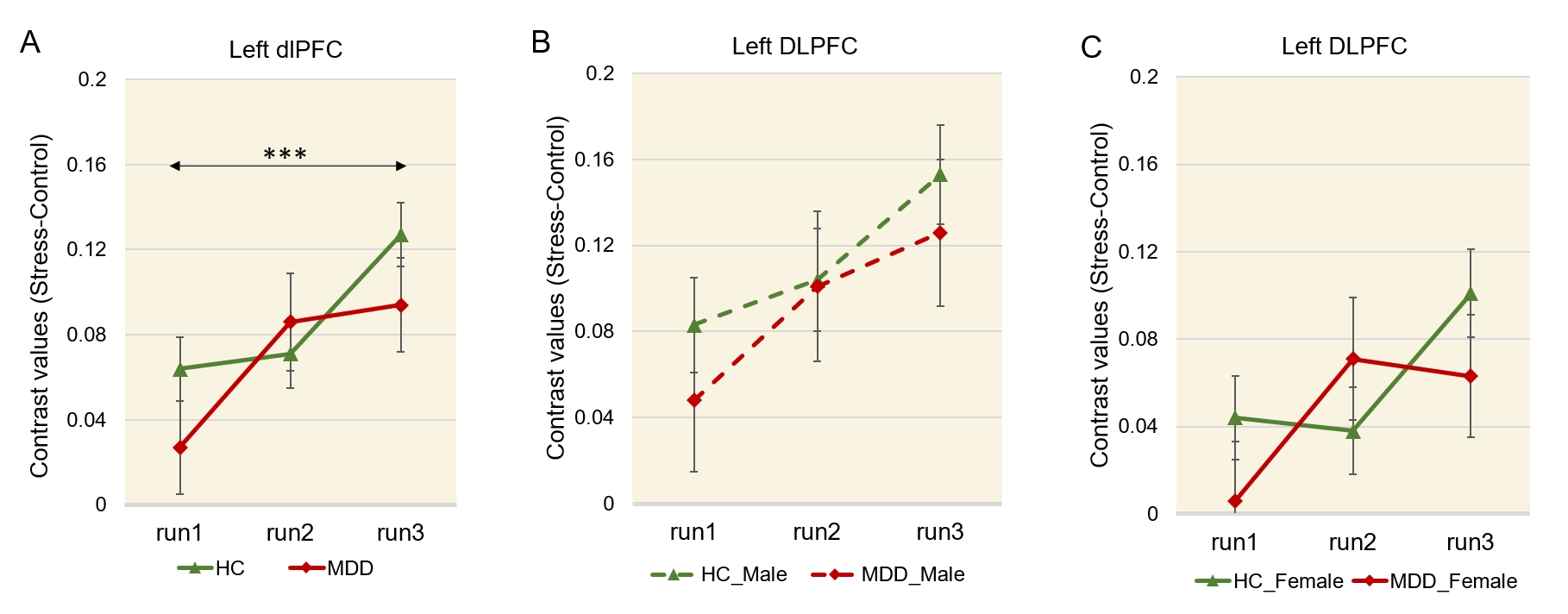


**Supplemental Fig. S3.** **Neural stress responses in the left dorsolateral prefrontal cortex in different runs.** **(A)** A significant main effect of *Time* emerged for the left dlPFC across all subjects. Graph **(B)** and **(C)** were plotted for displaying the neural patterns of left dlPFC over stress exposure time in the males and females. Estimated-mean are plotted, and error bars represents SE. HC: healthy controls; MDD: major depressive disorder; dlPFC: dorsolateral prefrontal cortex. ^***^*p* < 0.001


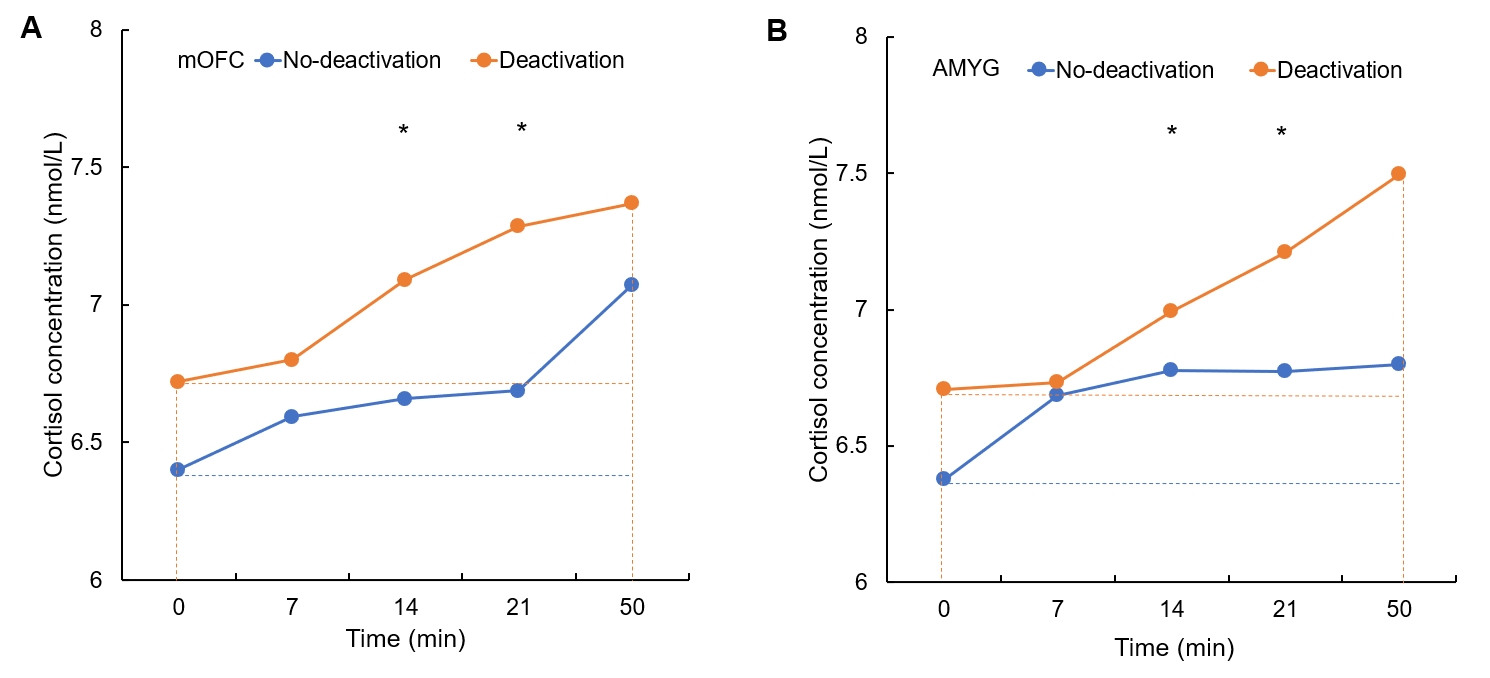


**Supplemental Fig. S4. Cortisol trajectory over the stress exposure in healthy females categorized by activation pattern of the medial orbitofrontal cortex (A) and amygdala (B).** Deactivation refers run3 < run1; no-deactivation refers to run3 > run1. mOFC: medial orbitofrontal cortex; AMYG: amygdala. ^*^ *p* < 0.05

**
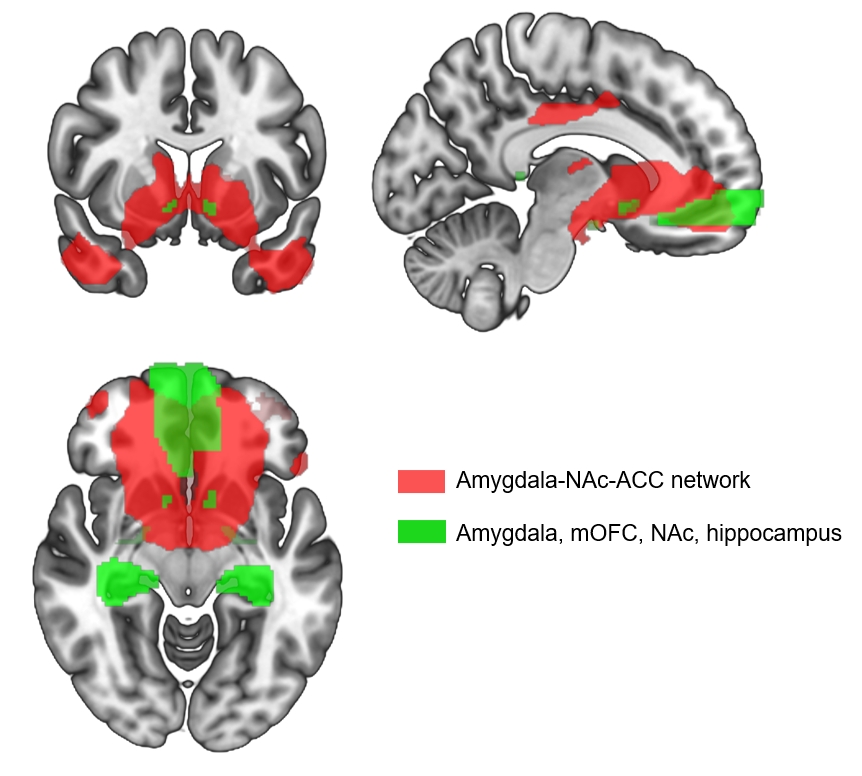
Supplemental Fig. S5. The amygdala-NAc-ACC network and the deactivated (Stress minus. control; amygdala, mOFC, NAc, hippocampus) regions of interest.** NAc: nucleus accumbens; ACC: anterior cingulate cortex; mOFC: medial orbitofrontal cortex

**Supplemental Tables**

**Supplemental Table S1:** Results of repeated-measures general linear model on cortisol and self-report responses to stress.

|  | **Cortisol Concentration** | |  | **Subjective Stress Rating** | |
| --- | --- | --- | --- | --- | --- |
|  | ***F-value*** | ***p-value*** |  | ***F-value*** | ***p-value*** |
| ***Within-subject effect*** |  |  |  |  |  |
| ***Time*** | **15.07** | **< 0.001** |  | **151.87** | **< 0.001** |
| *Time × Age* | 0.85 | 0.549 |  | 0.79 | 0.375 |
| *Time × Diagnosis* | 1.21 | 0.296 |  | 0.46 | 0.496 |
| *Time × Sex* | 0.45 | 0.873 |  | 0.28 | 0.599 |
| *Time × Diagnosis × Sex* | 1.02 | 0.418 |  | 0.001 | 0.972 |
| ***Between-subject effect*** |  |  |  |  |  |
| *Diagnosis* | 1.20 | 0.274 |  | 2.23 | 0.136 |
| *Sex* | 2.47 | 0.117 |  | 3.52 | 0.061 |
| *Diagnosis × Sex* | 0.28 | 0.598 |  | 1.24 | 0.266 |
| *Age* | < 0.001 | 0.995 |  | **8.26** | **0.004** |

**Supplemental Table S2.** Results of *Hemisphere* × *Time* × *Sex* × *Diagnosis* ANCOVA analyses in medial orbitofrontal cortex, amygdala, hippocampus, nucleus accumbens, and dorsolateral prefrontal cortex.

|  | **F-value (*p*-value)** | | | | |
| --- | --- | --- | --- | --- | --- |
|  | **mOFC** | **Amygdala** | **Hippocampus** | **NAc** | **dlPFC** |
| ***Within-subject effect*** |  |  |  |  |  |
| *Time* | **3.37 (0.035)** | 2.76 (0.064) | 0.91 (0.405) | 2.00 (0.136) | 2.59 (0.076) |
| *Time × Age* | 2.69 (0.069) | 1.11 (0.331) | 0.65 (0.525) | 1.73 (0.179) | 0.11 (0.899) |
| *Time* × *Diagnosis* | **3.21 (0.041)** | 0.25 (0.779) | 0.05 (0.956) | 1.09 (0.335) | <0.001 (0.999) |
| *Time* × *Sex* | 1.02 (0.362) | 1.61 (0.194) | 0.95 (0.386) | 0.844(0.431) | 0.06 (0.938) |
| *Time* × *Diagnosis* × *Sex* | **4.62 (0.010)** | **6.44 (0.002)** | 2.03 (0.133) | **4.77 (0.009)** | 1.76 (0.172） |
| *Hemisphere* | **19.96 (< 0.001)** | 0.02 (0.876) | 0.48 (0.489) | 0.31 (0.579) | **4.93 (0.027)** |
| *Hemisphere* × *Age* | 0.90 (0.344) | 0.16 (0.686) | 1.53 (0.217) | 0.69 (0.407) | 0.34 (0.562) |
| *Hemisphere* × *Diagnosis* | 1.94 (0.165) | 1.21 (0.273) | 0.66 (0.416) | 0.86 (0.355) | 0.17 (0.685) |
| *Hemisphere* × *Sex* | 0.16 (0.692) | 2.85 (0.092) | 1.40 (0.238) | **4.96 (0.027)** | 0.36 (0.550) |
| *Hemisphere* × *Diagnosis* × *Sex* | 1.93 (0.166) | 0.23 (0.629) | 0.56 (0.455) | 2.03 (0.156) | 0.03 (0.864) |
| *Time* × *Hemisphere* | 0.27 (0.767) | 0.28 (0.775) | 1.72 (0.180) | 0.14 (0.872) | **8.22 (< 0.001)** |
| *Time* × *Hemisphere × Age* | 0.69 (0.501) | 0.07 (0.937) | 0.17 (0.841) | 0.08 (0.925) | 1.26 (0.286) |
| *Time* × *Hemisphere* × *Diagnosis* | 1.29 (0.277) | 2.06 (0.128) | 0.68 (0.505) | 0.27 (0.767) | **4.64 (0.010)** |
| *Time* × *Hemisphere* × *Sex* | 0.75 (0.471) | 1.56 (0.212) | 0.25 (0.781) | 0.40 (0.671) | 0.73 (0.483) |
| *Time* × *Hemisphere* × *Diagnosis* × *Sex* | 0.47 (0.627) | 0.54 (0.581) | 0.67 (0.514) | 1.30 (0.274) | 2.12 (0.120) |
|  |  |  |  |  |  |
| ***Between-subject effect*** |  |  |  |  |  |
| *Age* | **4.26 (0.040)** | 2.97 (0.086) | 2.02 (0.157) | 1.25 (0.264) | 0.02 (0.882) |
| *Diagnosis* | 1.66 (0.199) | 0.88 (0.349) | 0.42 (0.515) | 0.46 (0.500) | 1.23 (0.268) |
| *Sex* | 0.75 (0.388) | 0.99 (0.320) | 0.88 (0.348) | 0.89 (0.347) | **8.43 (0.004)** |
| *Diagnosis* × *Sex* | 1.52 (0.219) | **5.76 (0.017)** | **4.73 (0.030)** | **4.76 (0.030)** | 0.01 (0.914) |

*Note. mOFC, medial orbitofrontal cortex; NAc, nucleus accumbens; dlPFC, dorsolateral prefrontal cortex*

**Supplemental Table S3.** Results of chi-square analyses in females.

| **Regions** | **Female MDD** | | **Female HC** | | **χ²** | ***P*** |
| --- | --- | --- | --- | --- | --- | --- |
|  | **run3 > run1** | **run3 < run1** | **run3 > run1** | **run3 < run1** |  |  |
| mOFC | 41 | 35 | 53 | 84 | 4.02 | **0.044** |
| amygdala | 42 | 34 | 50 | 87 | 6.27 | **0.012** |
| NAc | 46 | 30 | 59 | 78 | 5.28 | **0.021** |

*Note. mOFC, medial orbitofrontal cortex; NAc, nucleus accumbens; MDD, major depressive disorder; HC, healthy controls.*

**Supplemental Table S4.** MANCOVA results of four limbic-paralimbic-striatal regions (i.e., medial orbitofrontal cortex, amygdala, hippocampus, nucleus accumbens).

|  | **Wilks' Lambda** | **F** | ***df*** | ***P*** |
| --- | --- | --- | --- | --- |
| ***Within-subject effect*** |  |  |  |  |
| ***Region*** | **0.627** | **71.27** | **3, 360** | **< 0.001** |
| *Region × Age* | 0.986 | 1.67 | 3, 360 | 0.174 |
| *Region × Diagnosis* | 0.995 | 0.66 | 3, 360 | 0.579 |
| *Region × Sex* | 0.997 | 0.31 | 3, 360 | 0.820 |
| *Region × Diagnosis × Sex* | 0.984 | 2.01 | 3, 360 | 0.112 |
| *Hemisphere* | 0.995 | 1.93 | 1, 362 | 0.166 |
| *Hemisphere × Age* | 1.000 | 0.05 | 1, 362 | 0.818 |
| *Hemisphere × Diagnosis* | 0.991 | 3.13 | 1, 362 | 0.078 |
| ***Hemisphere × Sex*** | **0.983** | **6.14** | **1, 362** | **0.014** |
| *Hemisphere × Diagnosis × Sex* | 0.991 | 3.40 | 1, 362 | 0.066 |
| *Time* | 0.984 | 2.97 | 2, 361 | 0.052 |
| *Time × Age* | 0.989 | 2.04 | 2, 361 | 0.132 |
| *Time × Diagnosis* | 0.992 | 1.53 | 2, 361 | 0.217 |
| *Time × Sex* | 0.993 | 1.28 | 2, 361 | 0.279 |
| ***Time × Diagnosis × Sex*** | **0.970** | **5.61** | **2, 361** | **0.004** |
| ***Region × Hemisphere*** | **0.947** | **6.70** | **3, 360** | **< 0.001** |
| *Region × Hemisphere × Age* | 0.995 | 0.57 | 3, 360 | 0.636 |
| *Region × Hemisphere × Diagnosis* | 0.995 | 0.57 | 3, 360 | 0.637 |
| *Region × Hemisphere × Sex* | 0.986 | 1.66 | 3, 360 | 0.176 |
| *Region × Hemisphere × Diagnosis × Sex* | 0.995 | 0.65 | 3, 360 | 0.582 |
| *Region × Time* | 0.977 | 1.39 | 6, 357 | 0.219 |
| *Region × Time × Age* | 0.985 | 0.91 | 6, 357 | 0.488 |
| *Region × Time × Diagnosis* | 0.981 | 1.13 | 6, 357 | 0.344 |
| *Region × Time × Sex* | 0.991 | 0.51 | 6, 357 | 0.800 |
| ***Region × Time × Diagnosis × Sex*** | **0.942** | **3.64** | **6, 357** | **0.002** |
| *Hemisphere × Time* | 0.997 | 0.49 | 2, 361 | 0.615 |
| *Hemisphere × Time × Age* | 0.999 | 0.10 | 2, 361 | 0.909 |
| *Hemisphere × Time × Diagnosis* | 0.997 | 0.52 | 2, 361 | 0.598 |
| *Hemisphere × Time × Sex* | 0.999 | 0.25 | 2, 361 | 0.776 |
| *Hemisphere × Time × Diagnosis × Sex* | 0.996 | 0.46 | 2, 361 | 0.466 |
| *Region × Hemisphere × Time* | 0.992 | 0.46 | 6, 357 | 0.962 |
| *Region × Hemisphere × Time × Age* | 0.995 | 0.28 | 6, 357 | 0.945 |
| *Region × Hemisphere × Time × Diagnosis* | 0.981 | 1.18 | 6, 357 | 0.315 |
| *Region × Hemisphere × Time × Sex* | 0.980 | 1.18 | 6, 357 | 0.314 |
| *Region × Hemisphere × Time × Diagnosis × Sex* | 0.981 | 1.14 | 6, 357 | 0.337 |
|  |  |  |  |  |
| ***Between-subject effect*** |  |  |  |  |
| *Diagnosis* |  | 1.09 | 1, 362 | 0.298 |
| *Sex* |  | 1.06 | 1, 362 | 0.303 |
| ***Diagnosis × Sex*** |  | **4.41** | **1, 362** | **0.037** |
| *Age* |  | 3.10 | 1, 362 | 0.079 |

**Supplemental Table S5.** Results of the repeated measures of ANCOVA analyses in intra-network measures.

|  | **F-value (*p-value*)** | | |
| --- | --- | --- | --- |
|  | **Right FPN** | **Left FPN** | **Amygdala-NAc-ACC** |
| **Within-subject effect** |  |  |  |
| *Time* | 1.35 (0.261) | 0.38 (0.687) | **3.73 (0.025)** |
| *Time* × *Age* | 0.23 (0.798) | 0.07 (0.930) | 2.94 (0.054) |
| *Time* × *Diagnosis* | 0.48 (0.620) | 0.21 (0.813) | **4.67 (0.010)** |
| *Time* × *Sex* | 1.73 (0.177) | 0.08 (0.928) | 1.06 (0.349) |
| *Time* × *Diagnosis* × *Sex* | 1.17 (0.310) | 0.20 (0.821) | 1.11 (0.331) |
| **Between-subject effect** |  |  |  |
| *Diagnosis* | 2.36 (0.126) | 1.34 (0.248) | 2.36 (0.125) |
| *Sex* | **9.28 (0.002)** | 0.12 (0.734) | 0.43 (0.511) |
| *Diagnosis* × *Sex* | 1.32 (0.251) | 1.40 (0.237) | **5.70 (0.017)** |
| *Age* | 1.11 (0.292) | 0.27 (0.601) | 1.47 (0.226) |

*Note. FPN: frontoparietal network; NAc: nucleus accumbens; ACC: anterior cingulate cortex*

**Supplemental Table S6.** Correlations between neural, cortisol and questionnaire measures.

| Healthy females | | |
| --- | --- | --- |
|  | *AUC_g_*, *r(p)* | AUC_i_, *r(p)* |
| Amygdala (run3 minus run1) | **- 0.23 (0.014)** | - 0.07 (0.477) |
| mOFC (run3 minus run1) | **- 0.23 (0.013)** | - 0.06 (0.542) |
| NAc (run3 minus run1) | - 0.15 (0.117) | - 0.02 (0.874) |
|  | | |
| MDD patients | | |
|  | *AUC_g_*, *r(p)* | *AUC_i_*, *r(p)* |
| Amygdala-NAc-ACC network (run3 minus run1) | 0.03 (0.751) | 0.07 (0.460) |
|  | *BDI,* *r(p)* |  |
|  | - 0.01(0.892) |  |
|  | | |
| MDD males | | |
|  | *BDI, r(p)* |  |
| Amygdala | 0.02 (0.912) |  |
| Hippocampus | 0.03 (0.853) |  |
| NAc | - 0.02 (0.915) |  |
| mOFC | - 0.03 (0.855) |  |
| dlPFC | - 0.13 (0.392) |  |
| Right frontal parietal network | 0.06 (0.704) |  |
| Left frontal parietal network | - 0.05 (0.722) |  |
| Amygdala-NAc-ACC network | 0.11 (0.459) |  |
|  | | |
| MDD females | | |
|  | *BDI, r(p)* |  |
| Amygdala | - 0.21 (0.075) |  |
| Hippocampus | **- 0.29 (0.011)** |  |
| NAc | - 0.10 (0.384) |  |
| mOFC | - 0.13 (0.280) |  |
| dlPFC | 0.004 (0.972) |  |
| Right frontal parietal network | - 0.03 (0.804) |  |
| Left frontal parietal network | 0.02 (0.875) |  |
| Amygdala-NAc-ACC network | - 0.13 (0.259) |  |

*Note. AUC_g_, area under curve with regard to ground; AUC_i_, area under curve with regard to increase; mOFC, medial orbital frontal cortex; NAc, nucleus accumbens; ACC, anterior cingulate cortex; dlPFC, dorsolateral prefrontal cortex.*

**Supplemental Table S7. Correlation matrix among all variables in healthy males**

| **(1) mOFC** | - |  |  |  |  |  |  |  |  |  |  |  |  |  |  |  |
| --- | --- | --- | --- | --- | --- | --- | --- | --- | --- | --- | --- | --- | --- | --- | --- | --- |
| **(2) Amygdala** | **0.78^**^** | - |  |  |  |  |  |  |  |  |  |  |  |  |  |  |
| **(3) NAc** | **0.68^***^** | **0.65^***^** | - |  |  |  |  |  |  |  |  |  |  |  |  |  |
| **(4) Hippocampus** | **0.63^***^** | **0.83^***^** | **0.48^***^** | - |  |  |  |  |  |  |  |  |  |  |  |  |
| **(5) dlPFC** | **0.29^**^** | **0.42^***^** | 0.13 | **0.59^***^** | - |  |  |  |  |  |  |  |  |  |  |  |
| **(6) left FPN** | 0.12 | 0.11 | -0.01 | **0.22^*^** | **0.39^***^** | - |  |  |  |  |  |  |  |  |  |  |
| **(7) right FPN** | 0.15 | 0.13 | -0.003 | 0.11 | **0.37^***^** | **0.48^***^** | - |  |  |  |  |  |  |  |  |  |
| **(8) Amy-NAc-ACC network** | **0.61^***^** | **0.61^***^** | **0.78^***^** | **0.38^***^** | -0.002 | -0.18 | -0.14 | - |  |  |  |  |  |  |  |  |
| **(9) NAc (run3-run1)** | -0.14 | -0.15 | -0.18 | -0.06 | -0.01 | -0.04 | 0.13 | **-0.21^*^** | - |  |  |  |  |  |  |  |
| **(10) mOFC (run3-run1)** | **-0.27^**^** | **-0.23^*^** | **-0.26^**^** | -0.13 | -0.12 | -0.12 | -0.03 | **-0.20^*^** | **0.64^***^** | - |  |  |  |  |  |  |
| **(11) Amygdala (run3-run1)** | **-0.27^**^** | **-0.20^*^** | **-0.25^*^** | **-0.20^*^** | -0.001 | -0.07 | 0.08 | **-0.20^*^** | **0.63^***^** | **0.61^***^** | - |  |  |  |  |  |
| **(12) Amy-NAc-ACC network (run3-run1)** | -0.15 | -0.10 | -0.16 | -0.04 | 0.04 | -0.09 | 0.08 | -0.15 | **0.64^***^** | **0.58^***^** | **0.46^***^** | - |  |  |  |  |
| **(13) BDI score** | 0.06 | 0.11 | 0.15 | 0.06 | 0.009 | 0.19 | -0.12 | 0.06 | **-0.29^**^** | -0.16 | **-0.25^**^** | **-0.24^*^** | - |  |  |  |
| **(14) AUCg** | -0.10 | -0.07 | -0.02 | 0.02 | -0.06 | -0.03 | -0.18 | 0.03 | 0.05 | 0.17 | 0.06 | 0.06 | -0.02 | - |  |  |
| **(15) AUCi** | -0.09 | -0.03 | 0.02 | -0.02 | 0.14 | -0.04 | 0.02 | -0.09 | 0.10 | 0.003 | 0.16 | **0.22^*^** | **-0.23^*^** | **0.22^*^** | - |  |
| **(16) Mean FD** | 0.17 | 0.19 | 0.14 | 0.17 | 0.11 | -0.12 | -0.16 | **0.21^*^** | 0.03 | 0.01 | 0.09 | 0.12 | -0.07 | 0.18 | -0.04 | - |
| **(17) Age** | **0.25^*^** | 0.09 | 0.07 | 0.08 | -0.02 | 0.06 | -0.05 | 0.05 | 0.005 | -0.02 | -0.04 | -0.08 | -0.02 | 0.01 | 0.03 | -0.07 |
|  | **(1)** | **(2)** | **(3)** | **(4)** | **(5)** | **(6)** | **(7)** | **(8)** | **(9)** | **(10)** | **(11)** | **(12)** | **(13)** | **(14)** | **(15)** | **(16)** |

*Note. mOFC, medial orbitofrontal cortex; NAc, nucleus accumbens; dlPFC, dorsolateral prefrontal cortex; FPN, frontoparietal network; Amy, amygdala, ACC, anterior cingulate cortex; BDI, Beck Depression Inventory; AUCg, area under curve with regard to ground; AUCi, area under the curve with regard to increase; FD, framewise displacement. ^*^ p < 0.05, ^**^ p < 0.01, ^***^ p < 0.001.*

Supplemental Table S8. Correlation matrix among all variables in healthy females.

| **(1) mOFC** | - |  |  |  |  |  |  |  |  |  |  |  |  |  |  |  |
| --- | --- | --- | --- | --- | --- | --- | --- | --- | --- | --- | --- | --- | --- | --- | --- | --- |
| **(2) Amygdala** | **0.69^***^** | - |  |  |  |  |  |  |  |  |  |  |  |  |  |  |
| **(3) NAc** | **0.75^***^** | **0.77^***^** | - |  |  |  |  |  |  |  |  |  |  |  |  |  |
| **(4) Hippocampus** | **0.56^***^** | **0.89^***^** | **0.63^***^** | - |  |  |  |  |  |  |  |  |  |  |  |  |
| **(5) dlPFC** | -0.03 | -0.02 | 0.008 | 0.09 | - |  |  |  |  |  |  |  |  |  |  |  |
| **(6) left FPN** | -0.05 | 0.06 | -0.02 | 0.11 | **0.27^**^** | - |  |  |  |  |  |  |  |  |  |  |
| **(7) right FPN** | -0.13 | **-0.18^*^** | **-0.18^*^** | -0.12 | **0.32^***^** | **0.32^***^** | - |  |  |  |  |  |  |  |  |  |
| **(8) Amy-NAc-ACC network** | **0.51^***^** | **0.55^***^** | **0.73^***^** | **0.41^***^** | 0.005 | -0.40 | **-0.23^**^** | - |  |  |  |  |  |  |  |  |
| **(9) NAc (run3-run1)** | **0.28^**^** | **0.24^**^** | **0.37^***^** | **0.18^*^** | 0.14 | -0.004 | 0.05 | **0.40^***^** | - |  |  |  |  |  |  |  |
| **(10) mOFC (run3-run1)** | **0.39^***^** | **0.23^**^** | **0.21^*^** | **0.20^*^** | 0.14 | -0.02 | -0.05 | **0.18^*^** | **0.59^***^** | - |  |  |  |  |  |  |
| **(11) Amygdala (run3-run1)** | 0.13 | 0.13 | 0.11 | 0.13 | -0.03 | -0.08 | 0.01 | **0.18^*^** | **0.63^***^** | **0.47^***^** | - |  |  |  |  |  |
| **(12) Amy-NAc-ACC network (run3-run1)** | 0.11 | 0.11 | 0.18^*^ | 0.09 | **0.19^*^** | 0.01 | 0.12 | **0.31^***^** | **0.62^***^** | **0.44^***^** | **0.40^***^** | - |  |  |  |  |
| **(13) BDI score** | 0.05 | 0.04 | 0.10 | 0.09 | -0.08 | 0.03 | -0.11 | 0.16 | 0.05 | -0.04 | -0.004 | 0.08 | - |  |  |  |
| **(14) AUCg** | -0.05 | -0.02 | -0.04 | -0.03 | 0.04 | **0.23^*^** | **0.22^*^** | -0.13 | -0.15 | **-0.23^*^** | **-0.23^*^** | -0.04 | 0.10 | - |  |  |
| **(15) AUCi** | 0.05 | 0.10 | 0.08 | 0.09 | 0.02 | 0.07 | 0.17 | 0.06 | -0.02 | -0.06 | -0.07 | 0.16 | 0.01 | **0.28^**^** | - |  |
| **(16) Mean FD** | -0.08 | -0.15 | 0.02 | -0.14 | **-0.18^*^** | 0.04 | -0.01 | 0.15 | 0.05 | -0.02 | 0.01 | 0.04 | 0.16 | 0.01 | 0.002 | - |
| **(17) Age** | 0.08 | 0.11 | 0.05 | 0.08 | -0.08 | 0.02 | < 0.001 | 0.06 | 0.02 | 0.02 | 0.06 | -0.02 | -0.08 | 0.09 | 0.12 | 0.16 |
|  | **(1)** | **(2)** | **(3)** | **(4)** | **(5)** | **(6)** | **(7)** | **(8)** | **(9)** | **(10)** | **(11)** | **(12)** | **(13)** | **(14)** | **(15)** | **(16)** |

*Note. mOFC, medial orbitofrontal cortex; NAc, nucleus accumbens; dlPFC, dorsolateral prefrontal cortex; FPN, frontoparietal network; Amy, amygdala, ACC, anterior cingulate cortex; BDI, Beck Depression Inventory; AUCg, area under curve with regard to ground; AUCi, area under the curve with regard to increase; FD, framewise displacement. ^*^ p < 0.05, ^**^ p < 0.01, ^***^ p < 0.001.*

Supplemental Table S9. Correlation matrix among all variables in depressed males.

| **(1) mOFC** | - |  |  |  |  |  |  |  |  |  |  |  |  |  |  |  |
| --- | --- | --- | --- | --- | --- | --- | --- | --- | --- | --- | --- | --- | --- | --- | --- | --- |
| **(2) Amygdala** | **0.82^***^** | - |  |  |  |  |  |  |  |  |  |  |  |  |  |  |
| **(3) NAc** | **0.80^***^** | **0.84^***^** | - |  |  |  |  |  |  |  |  |  |  |  |  |  |
| **(4) Hippocampus** | **0.78^***^** | **0.94^***^** | **0.79^***^** | - |  |  |  |  |  |  |  |  |  |  |  |  |
| **(5) dlPFC** | **0.34^*^** | 0.14 | 0.14 | 0.18 | - |  |  |  |  |  |  |  |  |  |  |  |
| **(6) left FPN** | 0.24 | 0.06 | 0.14 | 0.06 | **0.62^***^** | - |  |  |  |  |  |  |  |  |  |  |
| **(7) right FPN** | 0.16 | -0.04 | -0.01 | -0.02 | **0.67^***^** | **0.54^***^** | - |  |  |  |  |  |  |  |  |  |
| **(8) Amy-NAc-ACC network** | **0.61^***^** | **0.68^***^** | **0.76^***^** | **0.58^***^** | -0.06 | -0.07 | -0.19 | - |  |  |  |  |  |  |  |  |
| **(9) NAc (run3-run1)** | **0.39^**^** | **0.36^*^** | **0.48^**^** | **0.40^**^** | **0.29^*^** | 0.24 | **0.33^*^** | 0.28 | - |  |  |  |  |  |  |  |
| **(10) mOFC (run3-run1)** | **0.55^***^** | **0.44^**^** | **0.43^**^** | **0.46^**^** | **0.38^**^** | 0.26 | **0.41^**^** | 0.25 | **0.74^***^** | - |  |  |  |  |  |  |
| **(11) Amygdala (run3-run1)** | **0.37^*^** | **0.36^*^** | **0.43^**^** | **0.38^**^** | 0.24 | 0.17 | 0.24 | 0.36 | **0.83^***^** | **0.75^***^** | - |  |  |  |  |  |
| **(12) Amy-NAc-ACC network (run3-run1)** | **0.30^*^** | **0.34^*^** | 0.27 | **0.40^**^** | 0.14 | 0.01 | 0.04 | 0.16 | **0.62^***^** | **0.54^***^** | **0.52^***^** | - |  |  |  |  |
| **(13) BDI score** | -0.03 | 0.02 | -0.02 | 0.03 | -0.13 | -0.05 | 0.06 | 0.11 | -0.01 | 0.08 | 0.06 | -0.05 | - |  |  |  |
| **(14) AUCg** | **-0.34^*^** | **-0.32^*^** | -0.25 | **-0.32^*^** | 0.12 | 0.09 | 0.15 | -0.07 | -0.02 | -0.06 | 0.14 | 0.004 | 0.08 | - |  |  |
| **(15) AUCi** | -0.19 | **-0.19^*^** | -0.11 | -0.14 | -0.12 | 0.007 | 0.04 | -0.25 | 0.17 | 0.02 | 0.08 | 0.05 | 0.13 | 0.11 | - |  |
| **(16) Mean FD** | 0.19 | 0.26 | 0.13 | 0.22 | -0.13 | -0.06 | -**0.37^*^** | 0.19 | -0.07 | 0.005 | -0.17 | 0.03 | -0.12 | -0.05 | -0.16 | - |
| **(17) Age** | 0.09 | -0.16 | -0.09 | -0.15 | 0.18 | 0.17 | 0.08 | -0.04 | -0.11 | -0.07 | -0.10 | -0.28 | 0.15 | 0.14 | -0.14 | -0.03 |
|  | **(1)** | **(2)** | **(3)** | **(4)** | **(5)** | **(6)** | **(7)** | **(8)** | **(9)** | **(10)** | **(11)** | **(12)** | **(13)** | **(14)** | **(15)** | **(16)** |

*Note. mOFC, medial orbitofrontal cortex; NAc, nucleus accumbens; dlPFC, dorsolateral prefrontal cortex; FPN, frontoparietal network; Amy, amygdala, ACC, anterior cingulate cortex; BDI, Beck Depression Inventory; AUCg, area under curve with regard to ground; AUCi, area under the curve with regard to increase; FD, framewise displacement. ^*^ p < 0.05, ^**^ p < 0.01, ^***^ p < 0.001.*

Supplemental Table S10. Correlation matrix among all variables in depressed females.

| **(1) mOFC** | - |  |  |  |  |  |  |  |  |  |  |  |  |  |  |  |
| --- | --- | --- | --- | --- | --- | --- | --- | --- | --- | --- | --- | --- | --- | --- | --- | --- |
| **(2) Amygdala** | **0.53^***^** | - |  |  |  |  |  |  |  |  |  |  |  |  |  |  |
| **(3) NAc** | **0.63^***^** | **0.84^***^** | - |  |  |  |  |  |  |  |  |  |  |  |  |  |
| **(4) Hippocampus** | **0.48^***^** | **0.89^***^** | **0.69^***^** | - |  |  |  |  |  |  |  |  |  |  |  |  |
| **(5) dlPFC** | 0.17 | 0.15 | 0.03 | 0.16 | - |  |  |  |  |  |  |  |  |  |  |  |
| **(6) left FPN network** | -0.11 | -0.14 | -0.12 | -0.11 | **0.47^***^** | - |  |  |  |  |  |  |  |  |  |  |
| **(7) right FPN network** | -0.10 | -0.14 | -0.15 | -0.16 | **0.51^***^** | **0.49^***^** | - |  |  |  |  |  |  |  |  |  |
| **(8) Amy-NAc-ACC network** | **0.51^***^** | **0.73^***^** | **0.87^***^** | **0.58^***^** | 0.02 | -0.08 | -0.18 | - |  |  |  |  |  |  |  |  |
| **(9) NAc (run3-run1)** | 0.14 | 0.03 | 0.12 | 0.05 | -0.22 | -0.09 | **-0.23^*^** | **0.24^*^** | - |  |  |  |  |  |  |  |
| **(10) mOFC (run3-run1)** | 0.04 | 0.19 | 0.20 | 0.20 | -0.08 | -0.11 | -0.20 | **0.31^**^** | **0.71^***^** | - |  |  |  |  |  |  |
| **(11) Amygdala (run3-run1)** | **0.28^*^** | 0.10 | 0.19 | 0.07 | 0.03 | 0.001 | -0.11 | **0.29^*^** | **0.62^***^** | **0.58^***^** | - |  |  |  |  |  |
| **(12) Amy-NAc-ACC network (run3-run1)** | 0.007 | 0.01 | 0.007 | 0.02 | -0.16 | -0.19 | -0.18 | 0.05 | **0.72^***^** | **0.64^***^** | **0.55^***^** | - |  |  |  |  |
| **(13) BDI score** | -0.13 | -0.21 | -0.10 | **-0.29^*^** | 0.004 | 0.02 | -0.03 | -0.13 | 0.04 | -0.02 | -0.10 | 0.02 | - |  |  |  |
| **(14) AUCg** | 0.003 | 0.04 | 0.05 | 0.07 | 0.06 | 0.09 | 0.17 | 0.11 | -0.03 | -0.10 | 0.02 | 0.05 | 0.005 | - |  |  |
| **(15) AUCi** | 0.08 | -0.01 | 0.02 | 0.07 | 0.04 | -0.09 | 0.02 | 0.007 | -0.01 | -0.19 | -0.08 | 0.09 | 0.04 | **0.45^***^** | - |  |
| **(16) Mean FD** | 0.05 | 0.06 | 0.03 | 0.006 | 0.14 | 0.07 | -0.02 | 0.06 | -0.20 | -0.05 | -0.08 | -0.27^*^ | 0.02 | -0.13 | -0.10 | - |
| **(17) Age** | 0.13 | **0.24^*^** | 0.20 | 0.20 | -0.003 | -0.04 | -0.17 | 0.16 | -0.22 | -0.31 | -0.15 | -0.21 | 0.03 | -0.02 | 0.09 | 0.06 |
|  | **(1)** | **(2)** | **(3)** | **(4)** | **(5)** | **(6)** | **(7)** | **(8)** | **(9)** | **(10)** | **(11)** | **(12)** | **(13)** | **(14)** | **(15)** | **(16)** |

*Note. mOFC, medial orbitofrontal cortex; NAc, nucleus accumbens; dlPFC, dorsolateral prefrontal cortex; FPN, frontoparietal network; Amy, amygdala, ACC, anterior cingulate cortex; BDI, Beck Depression Inventory; AUCg, area under curve with regard to ground; AUCi, area under the curve with regard to increase; FD, framewise displacement. ^*^ p < 0.05, ^**^ p < 0.01, ^***^ p < 0.001.*

**Supplemental References**

1. First MB, Spitzer RL, Gibbon M, Williams JBW. Structured clinical interview for DSM-IV-TR axis I disorders-patient edition (SCID-I/P, 11/2002 revision). New York: Biometrics Research Department, New York State Psychiatric Institute. 2002.

2. Beck AT, Steer RA, Brown G. Beck depression inventory–II. Psychological Assessment. 1996. 1996.

3. Hamilton M. The Hamilton rating scale for depression. Assessment of depression, Springer; 1986. p. 143–152.

4. Pruessner JC, Kirschbaum C, Meinlschmid G, Hellhammer DH. Two formulas for computation of the area under the curve represent measures of total hormone concentration versus time-dependent change. Psychoneuroendocrinology. 2003; 28:916–931.

5. Khoury JE, Gonzalez A, Levitan RD, Pruessner JC, Chopra K, Basile VS, et al. Summary cortisol reactivity indicators: Interrelations and meaning. Neurobiology of Stress. 2015; 2:34–43.

6. Esteban O, Markiewicz CJ, Blair RW, Moodie CA, Isik AI, Erramuzpe A, et al. fMRIPrep: a robust preprocessing pipeline for functional MRI. Nature Methods. 2019; 16:111–116.

7. Esteban O, Moodie CA, Isik AI. FMRIPrep, Software. Zenodo. 2018.

8. Gorgolewski K, Burns CD, Madison C, Clark D, Halchenko YO, Waskom ML, et al. Nipype: a flexible, lightweight and extensible neuroimaging data processing framework in python. Frontiers in Neuroinformatics. 2011; 5:13.

9. Gorgolewski KJ, Esteban O, Markiewicz CJ, Ziegler E, Ellis DG, Notter MP, et al. Nipype. Software. Zenodo. 2018..

10. Tustison NJ, Avants BB, Cook PA, Zheng Y, Egan A, Yushkevich PA, et al. N4ITK: improved N3 bias correction. IEEE Transactions on Medical Imaging. 2010; 29:1310–1320.

11. Avants BB, Epstein CL, Grossman M, Gee JC. Symmetric diffeomorphic image registration with cross-correlation: evaluating automated labeling of elderly and neurodegenerative brain. Medical Image Analysis. 2008; 12:26–41.

12. Zhang Y, Brady M, Smith S. Segmentation of brain MR images through a hidden Markov random field model and the expectation-maximization algorithm. IEEE Transactions on Medical Imaging. 2001; 20:45–57.

13. Dale AM, Fischl B, Sereno MI. Cortical surface-based analysis: I. Segmentation and surface reconstruction. Neuroimage. 1999; 9:179–194.

14. Klein A, Ghosh SS, Bao FS, Giard J, Häme Y, Stavsky E, et al. Mindboggling morphometry of human brains. PLoS Computational Biology. 2017; 13:e1005350.

15. Fonov VS, Evans AC, McKinstry RC, Almli CR, Collins DL. Unbiased nonlinear average age-appropriate brain templates from birth to adulthood. NeuroImage. 2009:S102.

16. Evans AC, Janke AL, Collins DL, Baillet S. Brain templates and atlases. Neuroimage. 2012; 62:911–922.

17. Wang S, Peterson DJ, Gatenby JC, Li W, Grabowski TJ, Madhyastha TM. Evaluation of field map and nonlinear registration methods for correction of susceptibility artifacts in diffusion MRI. Frontiers in Neuroinformatics. 2017; 11:17.

18. Huntenburg JM. Evaluating nonlinear coregistration of BOLD EPI and T1w images. 2014.

19. Treiber JM, White NS, Steed TC, Bartsch H, Holland D, Farid N, et al. Characterization and correction of geometric distortions in 814 diffusion weighted images. PloS One. 2016; 11:e0152472.

20. Greve DN, Fischl B. Accurate and robust brain image alignment using boundary-based registration. Neuroimage. 2009; 48:63–72.

21. Jenkinson M, Bannister P, Brady M, Smith S. Improved optimization for the robust and accurate linear registration and motion correction of brain images. Neuroimage. 2002; 17:825–841.

22. Cox RW, Hyde JS. Software tools for analysis and visualization of fMRI data. NMR in Biomedicine: An International Journal Devoted to the Development and Application of Magnetic Resonance In Vivo. 1997; 10:171–178.

23. Pruim RHR, Mennes M, van Rooij D, Llera A, Buitelaar JK, Beckmann CF. ICA-AROMA: A robust ICA-based strategy for removing motion artifacts from fMRI data. Neuroimage. 2015; 112:267–277.

24. Nickerson LD, Smith SM, Öngür D, Beckmann CF. Using dual regression to investigate network shape and amplitude in functional connectivity analyses. Frontiers in Neuroscience. 2017; 11:115.

25. Beckmann CF, Mackay CE, Filippini N, Smith SM. Group comparison of resting-state FMRI data using multi-subject ICA and dual regression. Neuroimage. 2009; 47:S148.

26. Aho K, Derryberry D, Peterson T. Model selection for ecologists: the worldviews of AIC and BIC. Ecology. 2014; 95:631–636.

27. Henckens MJAG, Klumpers F, Everaerd D, Kooijman SC, van Wingen GA, Fernández G. Interindividual differences in stress sensitivity: Basal and stress-induced cortisol levels differentially predict neural vigilance processing under stress. Social Cognitive and Affective Neuroscience. 2016; 11:663-673.

28. Veer IM, Oei NYL, Spinhoven P, van Buchem MA, Elzinga BM, Rombouts SARB. Endogenous cortisol is associated with functional connectivity between the amygdala and medial prefrontal cortex. Psychoneuroendocrinology. 2012; 37:1039-1047.
